# Characterization of the Interaction between Rfa1 and Rad24 in *Saccharomyces cerevisiae*

**DOI:** 10.1371/journal.pone.0116512

**Published:** 2015-02-26

**Authors:** Gunjan Piya, Erica N. Mueller, Heather K. Haas, Padmaja L. Ghospurkar, Timothy M. Wilson, Jaime L. Jensen, Christopher L. Colbert, Stuart J. Haring

**Affiliations:** 1 Department of Chemistry and Biochemistry, North Dakota State University, Fargo, ND, 58108, United States of America; 2 Interdisciplinary Program in Cellular and Molecular Biology, North Dakota State University, Fargo, ND, 58108, United States of America; University of Miami Miller School of Medicine, UNITED STATES

## Abstract

Maintaining the integrity of the genome requires the high fidelity duplication of the genome and the ability of the cell to recognize and repair DNA lesions. The heterotrimeric single stranded DNA (ssDNA) binding complex Replication Protein A (RPA) is central to multiple DNA processes, which are coordinated by RPA through its ssDNA binding function and through multiple protein-protein interactions. Many RPA interacting proteins have been reported through large genetic and physical screens; however, the number of interactions that have been further characterized is limited. To gain a better understanding of how RPA functions in DNA replication, repair, and cell cycle regulation and to identify other potential functions of RPA, a yeast two hybrid screen was performed using the yeast 70 kDa subunit, Replication Factor A1 (Rfa1), as a bait protein. Analysis of 136 interaction candidates resulted in the identification of 37 potential interacting partners, including the cell cycle regulatory protein and DNA damage clamp loader Rad24. The Rfa1-Rad24 interaction is not dependent on ssDNA binding. However, this interaction appears affected by DNA damage. The regions of both Rfa1 and Rad24 important for this interaction were identified, and the region of Rad24 identified is distinct from the region reported to be important for its interaction with Rfc2 5. This suggests that Rad24-Rfc2-5 (Rad24-RFC) recruitment to DNA damage substrates by RPA occurs, at least partially, through an interaction between the N terminus of Rfa1 and the C terminus of Rad24. The predicted structure and location of the Rad24 C-terminus is consistent with a model in which RPA interacts with a damage substrate, loads Rad24-RFC at the 5’ junction, and then releases the Rad24-RFC complex to allow for proper loading and function of the DNA damage clamp.

## Introduction

The coordination of processes that duplicate and maintain the genetic information of a cell is critical for the prevention of mutation and cellular disease. One of the factors necessary for both DNA replication and DNA repair is the heterotrimeric complex Replication Protein A (RPA) [1]. This eukaryotic complex binds to single-stranded DNA (ssDNA) produced from the unwinding of DNA during synthesis or from the removal or processing of a DNA lesion. In this fashion, RPA prevents reannealing of ssDNA, promotes nuclease protection of ssDNA intermediates, promotes the processing and restoration of DNA to a double-stranded DNA (dsDNA) form, and acts as a sensor of DNA damage [2–5].

It is clear that RPA functions through physical interactions and recruitment (direct and indirect) of other proteins critical for processing ssDNA intermediates. These other proteins include factors necessary to replicate DNA (*e*.*g*., MCM proteins, DNA polymerase α; [6,7]), to repair DNA (*e*.*g*. Sgs1, Dna2, Mre11, Rad52, XPA, XPF; [8–13]), and to establish checkpoint arrest (*e*.*g*., Mec1-Ddc2, Rad24-RFC, Ddc1-Rad17-Mec3; [14–17]). The above represent a few of the examples where further characterization of an interaction has been performed beyond identification.

As a sensor of DNA damage, RPA is critical for cell cycle checkpoint establishment [18,19]. Once bound to ssDNA generated during processing of a DNA lesion, RPA can then recruit in two major factors necessary for checkpoint establishment. One factor that is recruited is yeast Mec1-Ddc2 (homologous to human ATR-ATRIP) [20,21]. This complex acts as a checkpoint kinase necessary to activate downstream factors through phosphorylation. Both yeast and human RPA have been demonstrated to interact with yeast Mec1-Ddc2 and ATR-ATRIP, respectively [14,21]. Another factor that is recruited is the yeast Rad24-RFC complex (homologous to human Rad17-RFC) [15]. Rad24 substitutes for Rfc1 in the Rfc1-Rfc2-5 complex and is important in response to DNA damage [22]. Once recruited to these sites, Rad24-RFC can facilitate loading of the yeast checkpoint clamp Ddc1-Rad17-Mec3 (homologous to human Rad9-Rad1-Hus1; 9-1-1 complex) [15–17]. The clamp is necessary for downstream signaling to occur, including phosphorylation (activation) of yeast Mec1 (homologous to human ATR) and subsequent activation of yeast Rad53 (homologous to human Chk2) [17,23,24]. Once activated, Rad53 then phosphorylates a number of protein targets, including yeast Dun1, whose downstream actions result in arrest of the cell cycle [25,26].

One of the candidates identified multiple times in a yeast two-hybrid screen for factors interacting with yeast Replication Factor A1 (Rfa1) encoded the C-terminal 198 residues of Rad24. Purified Rad24 is recruited to ssDNA-dsDNA (5’) junctions, but only in the presence of purified RPA and in the context of a Rad24-RFC complex [15,16]. In the presence of a mutant form of yeast RPA in which the N-terminus of Rfa1 contains a charge-reversal mutation in the DNA binding cleft of DBD-F (*i*.*e*., *rfa1-t11*), Rad24-RFC could no longer load onto 5’ junctions [15]. Furthermore, recruitment of the yeast 9-1-1 complex was also ablated [16]. Taken together, this would indicate that RFA and Rad24-RFC interact with one another, and loading onto junction DNA is facilitated through the function of the N-terminus of Rfa1. However, the interaction had not been mapped within the Rad24-RFC complex, nor had the N-terminus of Rfa1 been directly identified as the interaction site. In this study, we provide evidence that the Rfa1 N-terminus is directly responsible for protein interaction, and that interaction is not dependent on the DNA state. We also map the region of Rad24 important for the interaction to its C-terminus. Furthermore, we demonstrate that interaction between Rfa1-Rad24 occurs during mitotic cell growth, but may be altered in response to DNA damage. Altered interaction does not appear to be due to post-translational modification of the Rad24 C-terminus or the Rfa2 N-terminus. Sequence alignment of Rad24 and Rfc1, along with the identification of the interacting regions of Rad24 and Rfa1 are indicative of an interaction that is independent of Rfc2-5 (RFC); however, these other subunits likely also participate through interactions with RFA in loading Rad24-RFC onto damaged DNA templates.

## Materials and Methods

### Strains and plasmids

Yeast strains and plasmids used in these studies are listed in S1 and S2 Tables, respectively. The strain used to test complementation of *rfa1* two-hybrid constructs is RMY122-A, a haploid isogenic derivative of RMY122-α [27], which contains both *rfa1Δ*::*TRP1* and *rfa2* Δ::*TRP1*. To test complementation and DNA damage resistance of *rad24* mutants, the strain RMY122-A-rad24Δ was used.

For yeast two-hybrid analysis, the yeast strains EGY188 or EGY48 [28,29] were used. Strains carrying *rfa2* N-terminal mutations were generated in the EGY48 background by two-step gene replacement. Briefly, plasmids pTMW2, pTMW3, or pTMW4 were digested with *Sna*BI to linearize each and target integration. The digested plasmids were transformed into EGY48, and transformants were selected on synthetic complete media (0.5% ammonium sulfate, 0.34% yeast nitrogen base without amino acids) containing dextrose (2%) and lacking uracil (SD-Ura). The integrants were then grown overnight in liquid YPD (1% yeast extract, 2% peptone, 2% dextrose) and plated onto media containing 0.8 μg/mL 5-fluoroorotic acid (SD+5-FOA). Genomic DNA from 5-FOA-resistant cells was isolated and PCR was performed using primers O-338 and O-339 (S3 Table) to identify candidates with correct integration of the *rfa2* mutant gene. Since the mutant forms of *rfa2* were cDNA forms, they lack the 108 base pair (bp) intron and are easily distinguished by size. Also, all PCR fragments were subsequently sequenced (Eton Bioscience) to verify proper integration of the *rfa2* mutant gene.

The plasmid vectors used for two-hybrid screening and analysis in this study are all derivatives of pEG202 (*P*_*ADH1*_-*lexA-BD*; bait) [29], pJG4-5 (*P*_*GAL*_-*B42-AD*; prey) [29], and pGAL-lexA (*P*_*GAL*_-*lexA-BD*; bait) [8]. Yeast Replication Factor A (RFA) subunit genes were cloned into pEG202 and pJG4-5 using *in vivo* homologous recombination cloning [30]. All primers used to amplify the RFA subunit genes are listed in S3 Table. Briefly, *RFA1*, *RFA2*, or *RFA3* were amplified by PCR using primers with 40 nucleotides (nt) of homologous sequence to the appropriate cloning vector on the 5’ end and 20 nt of complementary sequence to the desired RFA subunit gene on the 3’ end. pEG202 or pJG4-5 were digested with *Nco*I or *Eco*RI, respectively, to linearize each plasmid. The linearized vectors were co-transformed with the corresponding PCR-amplified RFA subunit gene into EGY48, and transformed cells were plated onto media lacking histidine (SD-His) or lacking tryptophan (SD-Trp) for cells transformed with pEG202 or pJG4-5, respectively. The resulting colonies, some containing recombined vectors, were then scraped from the plates, DNA (both genomic and plasmid) was isolated, and this DNA was electroporated into DH10B bacterial cells. The bacterial cells containing plasmids were selected for on LB plate media containing 100 μg/mL ampicillin (LB+Amp). Plasmid DNA was isolated from individual bacterial colonies and analyzed by diagnostic restriction digests. Plasmids with inserts were sequenced (Eton Bioscience). All bait plasmids containing *RFA1* gene fragments that encode for the individual domains of Rfa1 are derivatives of pGAL-lexA and were kindly provided by Susan Gasser [8]. Prey plasmids containing *RAD24* and *rad24-*Δ*C* were generated and verified in a similar fashion to the *RFA*-gene containing prey vectors.

To generate all mutant forms of *rad24* used for two-hybrid analysis in this study, the plasmid pGP2 (originally isolated in the screen), containing Rad24 amino acid (aa) residues 461-659, was used as a template. *In vitro* site-directed mutagenesis was performed using the appropriate mutagenic primer (S3 Table) to generate *rad24-*Δ*C1*, *rad24-*Δ*C2*, *rad24-*Δ*C3*, *rad24-650*,*652*,*654SSS→DDD*, *rad24-650*,*652*,*654SSS→AAA*, *rad24-S637D*, *rad24-S637A*, and *rad24-*Δ*coil*. All mutagenesis reactions were performed using *Phusion* High-Fidelity DNA polymerase (New England BioLabs), and mutant constructs were verified by diagnostic restriction digests and sequencing (Eton Bioscience). The *rfa1-t11* mutant Rfa1 bait plasmid (pENM21) was generated by *in vitro* site-directed mutagenesis as above, except pSJH101 was used as the template with the rfa1-t11-REV mutagenic primer. To test the biological significance of *rad24* mutations that disrupt interaction with Rfa1, *in vitro* site-directed mutagenesis was performed using pENM22 as a template. The resulting *rad24* mutant plasmids were verified by sequencing.

### Two-hybrid interaction screen and assay

The DupLEX-A Yeast Two-Hybrid System (Origene) and *S*. *cerevisiae* DupLEX-A Yeast Two-Hybrid Genomic DNA Library (Origene) were used to perform a yeast two-hybrid screen. Bait (pEG202) plasmids were constructed as described above. To test for auto-activation of the 6x*O*_*lexA*_-*LEU2* reporter gene in the absence of a prey vector (*i*.*e*., false-positive), each RFA subunit bait plasmid was transformed into EGY48 cells, and transformants were selected for on SD-His plates. Four independent colonies were patched onto SD-His plates and subsequently replica plated onto SD-His-Leu plates. Growth on SD-His-Leu plates indicated auto-activation.

Once the bait constructs were tested for auto-activation, the plasmid pGP1 (*lexA-rfa1-FLAB*) was co-transformed with pSH18-34 (8x*O*_*lexA*_-*lacZ* reporter) into EGY48 cells, and transformants were selected for by plating onto SD-His-Ura media. One colony was used to make competent cells, the transformation efficiency of the cells was measured, and the *S*. *cerevisiae* DupLEX-A Yeast Two-Hybrid Genomic DNA Library was transformed into these cells and selected on SD-His-Trp-Ura (SD-HTU) media. The resulting 1.6x10^6^ independent transformants were collected, titered, and 1.6x10^7^ cells (10-fold excess of original number of transformants) were plated onto diagnostic media containing 2% galactose (SG-His-Trp-Ura-Leu). Plates were incubated at 30°C, and starting on day four post-transformation, larger colonies were patched onto SD-HTU plates through day ten. These initial patch master plates were then replica plated onto both SD-His-Trp-Ura-Leu (SD-HTUL; negative growth control) and SG-His-Trp-Ura-Leu (SG-HTUL; retest) and grown at 30°C for 3–4 days. Patches that only grew on the SG-HTUL plates were repicked onto SD-HTU plates as secondary master plates and maintained for further characterization.

Prey plasmids were isolated from 100 patches and sequenced (Eton Bioscience) using the primer pJG4-5-UP-Sequence (S3 Table), which sequenced the 5’ end of the fusion junction for each insert. WU-BLAST2 (http://www.yeastgenome.org/cgi-bin/blast-sgd.pl) was utilized to determine the identity of each sequence and to determine the fusion junction, with respect to both base position in the gene and amino acid position in the protein.

To determine if interactions are affected by DNA damage, the SD-HTU secondary master plates were replica plated onto diagnostic media containing 0.024% methyl methanesulfonate (SG-HTUL+MMS) and compared to growth on SG-HTUL media. Prey plasmid DNA was isolated from an additional 36 patches that showed decreased growth (*i*.*e*., decreased reporter gene expression). These plasmids were sequenced and their identity determined as described previously.

### Beta-galactosidase activity measurement

Yeast two-hybrid interactions and identification of interacting regions between two proteins were determined by measuring expression of the 8x*O*_*lexA*_-*lacZ* reporter on plasmid pSH18-34 in EGY188 cells. Qualitative measurement of β-galactosidase expression was performed using replica plating of cells onto media containing 40 μg/mL 5-bromo-4-chloro-3-indolyl-β-D-galactopyranoside (SG-HTU+X-gal).

Quantitative measurements of β-galactosidase activity (dependent on reporter gene expression levels) were performed by measuring cleavage of *o*-nitrophenyl-β-D-galactosidase (ONPG) to produce *o*-nitrophenol (ONP). The ONP produced can be quantified by an absorbance assay. Briefly, cells were grown in SD-His-Trp-Ura overnight at 30°C. The next day, cells were subcultured into selective media containing 2% sodium acetate (SNaOAc-His-Trp-Ura) and grown at 30°C until they reached a concentration of ~1x10^7^ cells/mL. Galactose (2%) was then added to the media to induce expression of the prey (and in some cases the bait) fusion protein, and the cells were incubated for 5 hr at 30°C in an air shaker. After incubation, 10 mL of cells were collected, and β-galactosidase activity was measured using the method described by Guarente [31]. Quantitation was from at least three independent experiments using at least three independent colonies for each sample per experiment. The Miller unit values for each experiment were then normalized and reported as relative values compared to the normalizing control (*i*.*e*., the interaction between Rfa1 and the Rad24 C-terminus).

### DNA damage spot assays

RMY122-A-rad24Δ cells containing WT *RAD24*- or mutant *rad24*-expressing vectors were assayed by a spot assay on media containing various concentrations of different types of DNA damaging agents. Cells were grown in liquid YPD overnight (16-24 hr) at 30°C at 220 RPM. The next day, cell concentrations were determined, and an initial dilution of cells to 2.5x10^5^ cells/mL was made. Three-fold serial dilutions were performed, and 5 μL from the initial dilution and serial dilutions were spotted onto the following plates: YPD, YPD+0.0019-0.015% methyl methanesulfonate (MMS), YPD+0.5–5 μg/mL camptothecin (CPT), YPD+40–160 mM hydroxyurea (HU), YPD+4–7 μg/mL phleomycin (PHL), YPD exposed to 20–50 J/m^2^ ultraviolet (UV) light, SD-His-Leu-Ura, and YPG. Plates were incubated for 2-4 days at 30°C, and growth differences between the mutant and wild-type cells were documented.

### Sequence alignments and structure prediction

All sequence alignments shown were examined using T-COFFEE (http://tcoffee.crg.cat/apps/tcoffee/do:regular; [32]) with default settings. All known protein interactions with Rfa1, Rfa2, or Rfa3 were obtained using BioGRID (http://www.thebiogrid.org; [33,34]). All currently reported phosphorylation sites for Rad24 were obtained using PhosphoGRID (http://www.phosphogrid.org; [35,36].

Secondary structure predictions for Rad24 were obtained using Jpred3 (http://www.compbio.dundee.ac.uk/www-jpred/; [37,38]) and COILS (http://embnet.vital it.ch/software/COILS_form.html; [39]). Briefly, the amino acid sequence for Rad24 (YER173W) was retrieved from the *Saccharomyces* Genome Database (SGD; http://www.yeastgenome.org) and used as the input sequence for Jpred3 and COILS, and the default parameters were used for each program. Human Rad17 (NP_002864.1) was retrieved from NCBI and yeast Rad24 and Rfc1 (YOR217W) from SGD. The sequence alignment was displayed using T-COFFEE output in Clustal format or using ALINE (http://crystal.scb.uwa.edu.au/charlie/software/aline/; [40]). Structural modeling of Rad24 was performed using Phyre2 [41], HHpred [42], I-TASSER [43], and Raptor X [44].

## Results

### Rfa1 protein interactions identified by a yeast two-hybrid screen

A common means for understanding the function of a protein in a pathway or the mechanism of a protein’s function is to identify the factors with which it associates. This has been done for numerous factors, including yeast RPA, called Replication Factor A (RFA). Examination of known protein interactors with yeast Rfa1, Rfa2, or Rfa3 via BioGRID (http://www.thebiogrid.org) reveals 625 reported interactions, 380 of which are unique to a single subunit, and 59 of which show interaction with two or more subunits of the RFA complex. These interactors were identified through global mass spectrometry, two-hybrid analysis, and synthetic genetic screens. Most have not been further characterized beyond the identification of interaction. A two-hybrid assay was used to identify RFA-interacting factors and to help gain a consensus on which factors to further characterize. Bait plasmid constructs in which the lexA DNA binding domain (BD) is fused to the N-terminus of each RFA subunit (Rfa1, Rfa2, or Rfa3) were generated. Upon examination of each construct for auto-activation of our reporter gene (*LEU2*), only the BD-Rfa1 fusion did not activate transcription of the reporter gene on its own. This is not unexpected, as both human and mouse lexA-BD-Rpa2 and lexA-BD-Rpa3 constructs also show auto-activation in previous independent studies [6,45]. Because both BD-Rfa2 and BD-Rfa3 showed expression of the reporter gene and growth on diagnostic media even in the absence of an interacting partner, BD-Rfa1 was chosen to perform a yeast two-hybrid screen for interactors with RFA.

The yeast strain EGY48 containing six lexA operators (6x*O*_*lexA*_) upstream of the *LEU2* reporter gene was co-transformed with the BD-Rfa1 bait (pGP1), pSH18-34 (a plasmid containing 8x*O*_*lexA*_-*lacZ* reporter), and the *S*. *cerevisiae* DupLEX-A Yeast Two-Hybrid prey library (Origene). Transformants were collected, titered, and plated out onto SG-HTUL plates to select for cells expressing the *LEU2* reporter gene (*i*.*e*., putative interaction). These colonies were collected and patched onto SD-HTU plates and replica plated to SD-HTUL and SG-HTUL plates to verify that expression of the *LEU2* reporter gene only occurred in the presence of galactose. Upon retesting, prey plasmids were isolated from 100 independent colonies and sequenced to reveal each insert. Thirty-one unique interactions were identified initially. Of these, 23 were novel interactions (S4 Table) and 8 interactions had been previously reported (Table 1) and are accessible from BioGRID [33,34].

**Table 1:** Proteins identified in two-hybrid screen to interact with Rfa1-FLAB and previously identified to interact with RFA.

Rfa1 Interactor	Times Identified in Initial Screen	Times Identified in Damage Screen	lexA Fusion Junction (aa Residue #)	Previously Reported	Reported Interacting Subunit	Assay(s) Used^*a*^
Dna2	21	5	122	Yes	Rfa1, Rfa2, Rfa3	AC-MS; CoLoc; DR; PE; RC; SL; 2H
Sgs1	4	2	421	Yes	Rfa1	AC-WB; PE; RC; 2H
Ptc1	3	1	185	Yes	Rfa2	NG
Mph1	2		867	Yes	Rfa1	AC-MS; AC-WB
Rad24	1	12	461	Yes	Rfa1	NG
Mcm5	1		108	Yes	Rfa1	Co-Loc
Rfa1	1		65	Yes	Rfa1, Rfa2, Rfa3	2H; AC-MS; AC-WB; Co-Pur; RC
Sac6	1		202	Yes	Rfa1	AC-MS
Pol32		1	139	Yes	Rfa1, Rfa2	NG; PE

^*a*^ All assays previously used to identify RFA interactors are listed in BioGRID (http://thebiogrid.org).

Assay designations are as follows: AC-MS = Affinity Capture-Mass Spectrometry; AC-WB = Affinity Capture-Western Blotting; Co-Loc = Co-localization; Co-Pur = Co-purification; DR = Dosage Rescue; NG = Negative Genetic; PE = Phenotypic Enhancement; RC = Reconstituted Complex; SL = Synthetic Lethality; 2H = Two-Hybrid.

**Fig 1:**
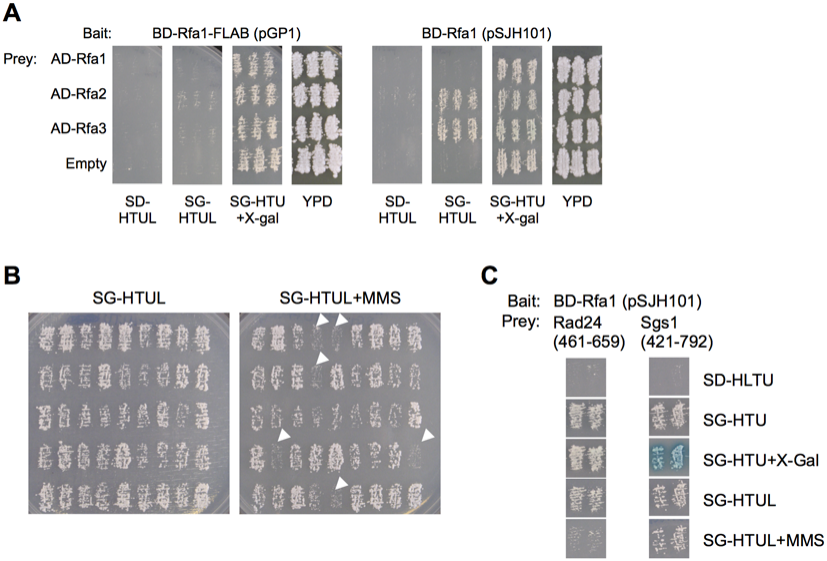
Two-hybrid screen, characterization of bait constructs used, and initial characterization of Rfa1-Rad24 interaction. [A] Interactions with Rfa2 and Rfa3. EGY48 (6x*O*_*lexA*_-*LEU2*) cells were co-transformed with pGP1 (BD-Rfa1-FLAB) or pSJH101 (BD-Rfa1) and pENM10 (B42-Rfa1), pENM11 (B42-Rfa2), or pENM12 (B42-Rfa3) and pSH18-34 (8x*O*_*lexA*_-*lacZ*). Nine independent transformants (three are shown) were subjected to replica plating onto SD-HTUL, SG-HTUL, YPD, and SG-HTU+X-gal plates. Growth on SG-HTUL and/or blue cells on SG-HTU+X-gal plates indicates interaction between the subunits. [B] Determining if an interaction is DNA-damage dependent. Colonies that indicated protein interaction with Rfa1 were patched to a SD-HTU plate, and this master plate was replica plated to SG-HTUL and SG-HTUL+MMS (methyl methanesulfonate), which induced DNA damage. Candidates that displayed dramatically reduced growth are denoted by white arrows. [C] Initial characterization of the interaction between Rfa1 and Rad24. The prey plasmids pGP2, encoding a Rad24 peptide (Rad24-ΔN) containing the C-terminal 198 amino acids (residues denoted in parentheses) or pGP3, encoding an Sgs1 peptide containing central amino acids 421-792 (denoted in parentheses), were co-transformed with pSJH101 (BD-Rfa1) and pSH18-34. Three independent colonies (two shown) were examined for interaction by measuring growth on SG-HTUL and blue color on SG-HTU+X-gal plates. The colonies were also examined for growth on SG-HTUL+MMS to indicate a damage-affected interaction. Sgs1 is a known interactor with Rfa1 [8].

Interestingly, none of the 100 sequenced candidates contained the other RFA subunits, Rfa2 or Rfa3. We generated prey (B42 activation domain; AD) constructs expressing AD-Rfa2 and AD-Rfa3 fusions. Tests of interactions between BD-Rfa1 and AD-Rfa2 or AD-Rfa3 revealed no expression of the reporter gene, indicating that these proteins did not interact (Fig. 1A; BD-Rfa1-FLAB (pGP1), see below). Upon resequencing of the BD-Rfa1 construct (and resequencing of the AD-Rfa2 and AD-Rfa3 constructs), it was revealed that the BD-Rfa1 construct contained a G→A base substitution at base position +1239 of Rfa1, generating a TGG→TAG nonsense mutation (leading to a truncated Rfa1 protein at tryptophan 413). This position lies very near the junction between DNA binding domain B (DBD-B) and DNA binding domain C (DBD-C) of Rfa1. Because the region missing (DBD-C) is normally responsible for RPA heterotrimerization, this truncated bait protein does not interact with the endogenous Rfa2 and Rfa3 subunits, and no functional RFA complex can be formed. In addition, we tested the complementation of this construct using plasmid shuffle and determined that the BD-Rfa1 fusion protein used for the screen (henceforth, referred to as BD-Rfa1-FLAB) was defective for cellular function (S1A Fig.). Although interactions identified using BD-Rfa1-FLAB cannot be in the context of an RFA complex, this tool was useful, because it indicates that an interaction with Rfa1-FLAB is not mediated through Rfa2 or Rfa3. Furthermore, this construct prevented oversaturation of our identified prey candidates with Rfa2 or Rfa3, which we have observed using full-length human BD-Rpa1 in an equivalent two-hybrid screen with an MCF7 cDNA library.

In addition to BD-Rfa1-FLAB, we generated two and acquired one full-length, sequence-verified BD-Rfa1 for all further characterization of identified BD-Rfa1-FLAB interactors. The full-length constructs are pPM07 [8], pSJH101, and pENM17 (S2 Table; pENM17 was not further utilized for interaction characterization due to very slight auto-activation). These constructs were tested for interaction with AD-Rfa2 and AD-Rfa3, and all three showed interactions with the other RFA subunits (Fig. 1A; S1B Fig.). Additionally, each was tested for complementation of *rfa1*Δ. Interestingly, pSJH101 and pENM17 were unable to complement the genomic deletion of *RFA1* (S1A Fig.), despite showing interactions with Rfa2 and Rfa3. However, the vector pPM07 did complement the deletion, but only in media containing dextrose, where expression of BD-Rfa1 is minimal due to leaky expression from the *GAL* promoter (S1A Fig.). pPM07 and pSJH101 encode otherwise identical lexA-fusion constructs, whereas pENM17 encodes for a lexA-fusion where the amino acid immediately upstream of the start codon is a leucine, instead of a histidine (Table 2). It appears that either constitutive expression from the *ADH1* promoter or overexpression from the *GAL* promoter is detrimental to BD-Rfa1 cellular function (*i*.*e*., it does not complement *rfa1*Δ). Additionally, overexpression of BD-Rfa1 from the *GAL* promoter prevents cell growth in cells that also express endogenous *RFA1* from its native promoter (*i*.*e*., dominant negative phenotype; S1A Fig., SG-HTU+X-gal when pPM07 is used). Therefore, pPM07 could not be used for a two-hybrid screen where growth of colonies is used to indicate interaction. The phenotypes of each bait construct are summarized in Table 2.

**Table 2:** Summary of BD-Rfa1 bait constructs.

					Rfa2/Rfa3 Interaction		
Plasmid Name	Promoter	Genotype	aa Adjacent to Rfa1 Start Codon	Complements *rfa1Δ*	SG-HTUL Growth	Color on X-gal	Rad24-ΔN460 Interaction
pGP1	ADH1	*rfa1-FLAB*	leucine	No	No	White	Yes
pPM07	GAL	*RFA1*	histidine	Yes (when not induced)	No	Dark Blue	Yes
pSJH101	ADH1	*RFA1*	histidine	No	Yes	Light blue	Yes
pENM17	ADH1	*RFA1*	leucine	No	Yes	Blue	Yes

### Abrogated interactions by treatment of cells with DNA damaging agents

The goal of the screen was not only to identify factors that interact with the RFA complex, but also to determine if these interactions are condition-dependent. In human cells, the RPA complex is post-translationally modified in response to DNA damaging agents, primarily in the form of hyper-phosphorylation of the N-terminus of Rpa2 on multiple serine/threonine residues [46,47]. It has been demonstrated that hyper-phosphorylation can lead to differential protein interaction with RPA [48], presumably to regulate RPA’s function in DNA metabolism in unstressed *vs*. stressed cells. To determine if our interactors displayed differential interactions with Rfa1 in response to DNA damage, we replicated our candidate patch master plates to media diagnostic for protein interactions in the absence (SG-HTUL) or presence of the DNA damaging agent methyl methanesulfonate (MMS). Because most of the interactors already displayed moderate-to-strong growth on diagnostic media, we could only confidently identify interactions that were reduced/eliminated, as determined by an inability to grow on diagnostic media containing MMS. Replica plating revealed that while most candidates retain their interaction on MMS-containing media, there were a noticeable number that do not (Fig. 1B). Upon prey DNA isolation and sequencing of 36 MMS-sensitive interaction candidates, it was identified that one-third (12/36) contained DNA encoding for the C-terminal 198 amino acids of Rad24 (Table 1). From the previous 100 randomly-selected candidates, we had only identified one candidate that encoded for Rad24. Furthermore, six additional candidates were identified, five of which were novel interactors (Table 1; S4 Table). The physiological relevance of each novel interactor has not yet been determined.

As the above was performed using originally identified interactors in the context of BD-Rfa1-FLAB, we tested the Rad24 interaction with a full-length BD-Rfa1 (pSJH101) in the context of unstressed or stressed (*i*.*e*., MMS-treated) cells. We also tested a candidate (pGP3) we had isolated independently from our screen that contained the region of Sgs1 that was characterized to interact with Rfa1 [8]. The C-terminal region of Rad24 identified also interacted with BD-Rfa1 in the context of an RFA complex, and this interaction was also reduced in the presence of methyl methanesulfonate (Fig. 1C). This is in contrast to Sgs1, where the interaction is not obviously negatively affected in the presence of DNA damage. Based on this result, we suggest that the Rfa1 and Rad24 interaction is strongly affected in response to DNA stress.

### Identifying the regions of Rfa1 and Rad24 that interact

Although there is previous evidence that RFA and Rad24-RFC interact, this evidence is primarily through the ability of purified recombinant RFA (or human RPA; all three subunits) to load purified recombinant Rad24-RFC (or human Rad17-RFC; five subunits) onto various DNA substrates *in vitro* [15,16,49]. Furthermore, it has been demonstrated that a purified recombinant mutant RFA complex containing *rfa1-t11* does not allow for Rad24-RFC loading onto DNA *in vitro* [15]. Also, Ddc1 (part of the yeast 9-1-1 complex) does not have the ability to localize to broken DNA in an *rfa1-t11* yeast strain [49]. Since the ability of Ddc1-Rad17-Mec3 to localize to a DNA break is dependent on Rad24-RFC, and the *rfa1-t11* mutation is localized to DBD-F of RFA/RPA, it was concluded that the N-terminus of Rfa1 is important to load Rad24-RFC (and subsequently, Ddc1-Rad17-Mec3) onto DNA. The importance of the Rpa1 N-terminus was also demonstrated using human proteins [17]. This suggests that Rfa1-Rad24-RFC interact or possibly that RFA/RPA binds the 5’ junction DNA and alters the substrate in a way that makes it more accessible to Rad24-RFC, although these may not be mutually exclusive. This also does not rule out the possibility that Rad24-RFC interacts with another region of Rfa1 or with the other RFA subunits. The two-hybrid assay provides a way to help make this distinction, because the two-hybrid assay is measuring the ability of proteins to interact independent of either the context of the DNA substrate (with the obvious exception that the lexA operator is on double-stranded DNA itself) or the context of the RFA complex. Although interactions with Rfa2 and Rfa3 could not be distinguished due to auto-activation and association with Rfa1, Rfa1 fragments could be used as bait, allowing for the identification of the region(s) of Rfa1 important for the interaction (assuming that interaction is not dependent on a particular DNA substrate).

**Fig 2:**
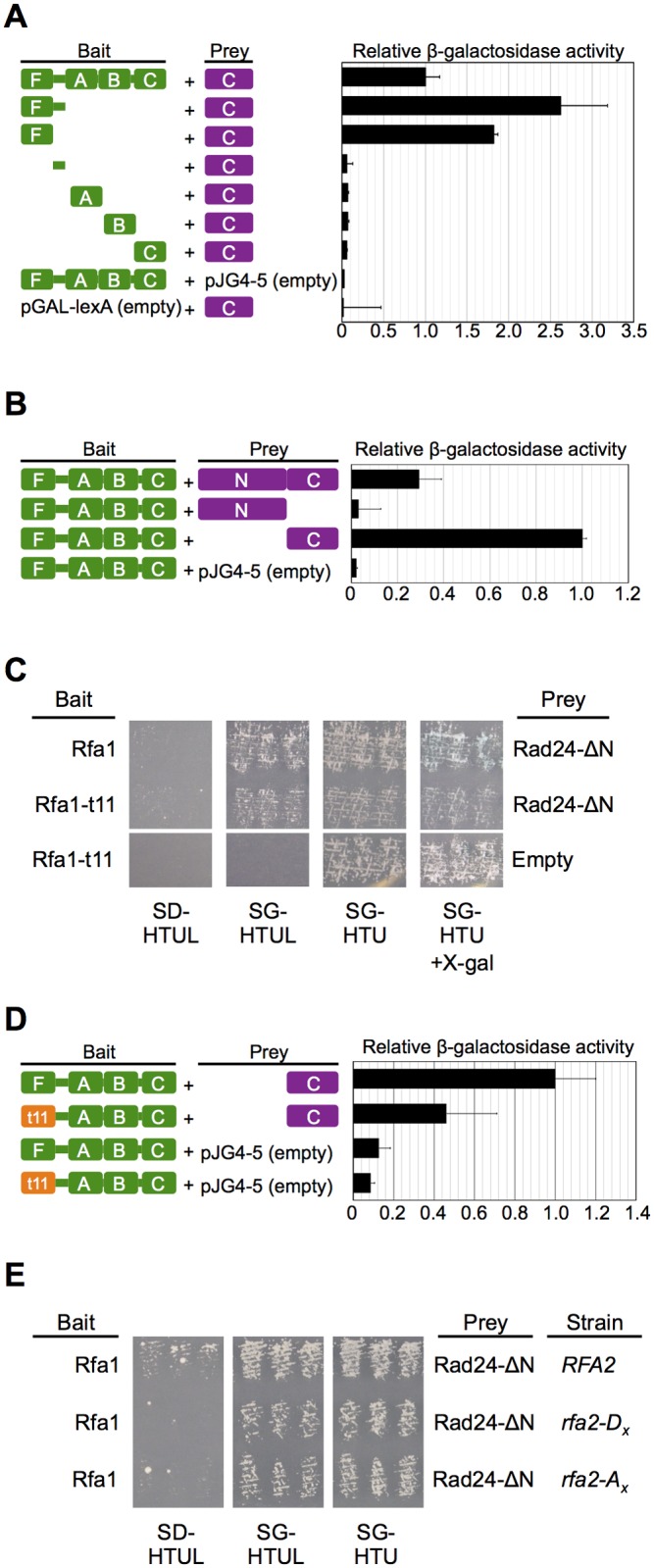
Mapping the regions important for the Rfa1-Rad24 interaction. [A] Rad24 interacts with the N-terminus of Rfa1. Rfa1 was divided into its major subdomains (DNA binding domains F, A, B, and C and/or the unstructured linker region between DBD-F and DBD-A) and expressed from a galactose-inducible promoter. The pGAL-lexA derivative bait plasmids were co-transformed into EGY188 (2x*O*_*lexA*_-*LEU2*) with pGP2 (AD-Rad24-ΔN) and pSH18-34. Liquid β-galactosidase assays were performed in at least three independent experiments using at least three independent transformants for each. β-galactosidase activity was normalized relative to the activity measured for the interaction between BD-Rfa1 (pPM07) and AD-Rad24-ΔN (pGP2). Empty bait or prey vectors were used as controls to ensure that BD-Rfa1 and AD-Rad24-ΔN did not display auto-activation. Standard error bars are shown. [B] Rfa1 interacts with the C-terminus of Rad24. Full-length AD-Rad24 and the N-terminal region (Rad24-ΔC; containing aa 1–460) not identified in the screen were examined to determine if the interaction is exclusively with the C-terminus of Rad24. The plasmids pGP17 (AD-Rad24), pGP18 (AD-Rad24-ΔC), or pGP2 (AD-Rad24-ΔN) were co-transformed with pPM07 (BD-Rfa1) and pSH18-34. β-galactosidase assays were performed and activity measured as in [A]. Again, values are normalized to the interaction between BD-Rfa1 and AD-Rad24-ΔN. [C] Examination of the Rfa1-t11 and Rad24 interaction. EGY48 cells were co-transformed with pSH18–34, pSJH101 (BD-Rfa1) or pENM21 (BD-Rfa1-t11), and pGP2 or pJG4–5 (empty prey vector). Nine independent transformants (three shown) were examined by replica plating on media diagnostic for interactions as in Fig. 1C. [D] Quantitation of the Rfa1-t11 and Rad24 interaction. The cells examined in [C] were also examined via a liquid β-galactosidase assay as described in [A], except that four independent transformants were examined in one experiment. Again, values were normalized relative to the activity measured for the interaction between BD-Rfa1 (pPM07) and AD-Rad24-ΔN (pGP2). [E] Examining whether the state of the Rfa2 N-terminus affects the Rfa1-Rad24 interaction. The endogenous *RFA2* in EGY48 was replaced with N-terminal *rfa2* mutations. *rfa2-D*_*x*_ and *rfa2-A*_*x*_ represent mutation of all serines/threonines contained within the first 34 amino acids of Rfa2 to aspartic acids or alanines, respectively, to mimic or prevent phosphorylation of the Rfa2 N-terminus. Phenotypes of these mutants in other strain backgrounds are described elsewhere [58] and are shown in S2 Fig. for the EGY48 background. Co-transformations into *rfa2* mutant strains were performed as designated, and interactions were assessed via replica plating. Three independent colonies are shown for each.

Hegnauer *et al*. [8] divided the Rfa1 protein into its individual DNA-binding domains (DBDs; based on human Rpa1 structural studies) and its intrinsically disordered region (linker) and cloned these into the bait vector pGAL-lexA (Fig. 2A; schematic for bait constructs). Using these constructs, we characterized the interaction region of Rfa1 with the Rad24 fragment we isolated from our screen by liquid beta-galactosidase assays. In addition to BD-Rfa1-FLAB interacting with the Rad24 C-terminus (S1 Fig.), full-length BD-Rfa1 (pPM07) also showed obvious beta-galactosidase activity when co-expressed with the Rad24 C-terminus (pGP2; Fig. 2A). Of the partitioned Rfa1 constructs, only Rfa1 protein fragments containing DBD-F (*i*.*e*., BD-Rfa1-DBD-F or BD-Rfa1-DBD-F+linker) showed beta-galactosidase activity (Fig. 2A). This is consistent with the previous observations that the Rfa1 N-terminus is necessary to load Rad24-RFC [15,49], but also provides evidence that Rfa1 can interact with Rad24 independent of single-stranded or junction DNA binding. This also indicated that other regions of Rfa1 do not play a significant role in the interaction. Because this interaction was identified outside of the context of an RFA complex, it also indicates that the association with DBD-F and does not require the other RFA subunits (although they might affect the strength of interaction). Although this interaction may be direct, bridging through proteins that interact with DBD-F cannot be ruled out by this assay.

By virtue of identifying Rad24 through a yeast two-hybrid genomic library, we already limited the interaction to the C-terminal 198 amino acids (aa 461-659) of Rad24. However, this does not preclude other regions of this protein from being important for interaction with Rfa1. To determine this, we cloned the full-length *RAD24* gene, as well as a *rad24* gene fragment that encoded for the first 460 aa (*rad24-*Δ*C*) into prey vectors (Fig. 2B). Beta-galactosidase activity was observed for BD-Rfa1 and the full-length AD-Rad24 or AD-Rad24-ΔN construct originally isolated, but not the AD-Rad24-ΔC protein fragment containing the N-terminal 460 aa of Rad24 (Fig. 2B). Therefore, we conclude that interacting region lies specifically within the C-terminal 198 aa of Rad24.

### The RFA-Rad24 interaction is affected by the context of the N-terminus of Rfa1, but not Rfa2

Given that the Rad24 C-terminus interacts with the Rfa1 N-terminus, and that *in vitro*, *rfa1-t11* prevents association of Rad24-RFC with a DNA substrate representing damaged DNA, we predicted that the *rfa1-t11* mutation should abrogate interaction between Rfa1 and Rad24. To test this, we generated BD-Rfa1-t11 (pENM21) and examined interaction with the C-terminus of Rad24 (Fig. 2C). Two observations were made. First, cells display a slight growth defect (Fig. 2C; SG-HTU), but only in the context of cells that express both BD-Rfa1-t11 and AD-Rad24-ΔN. This also shows as less growth on SG-HTUL and SG-HTU+X-gal. The second observation was that readily observable growth occurred on SG-HTUL and cells were still very light blue on SG-HTU+X-gal. To verify that β-galactosidase activity was indeed observable for cells expressing BD-Rfa1-t11 and AD-Rad24-ΔN, a quantitative liquid assay was performed. Fig. 2D demonstrates that the interaction is reduced, but still detectable between Rfa1-t11 and the Rad24 C-terminus. Again, this reduced interaction is consistent with the observed lack of loading of Rad24-RFC by RFA containing the Rfa1-t11 subunit [15,16]

Originally, we had wished to identify RFA interactors and determine if they were dependent on the state of the Rfa2 N-terminus. As the phospho-state of the human Rpa2 N-terminus appears to coordinate protein interactions with the human RPA complex, we predicted that the apparent “phospho-state” of the yeast Rfa2 N-terminus might also regulate yeast RFA interactions in response to DNA damage. Because all Rfa2 bait constructs auto-activate the reporter gene, a different approach was used. The native *RFA2* gene in EGY48 cells was replaced with one of two mutant forms of *rfa2*, making each mutant form the only form of Rfa2 protein expressed in the cell. These mutant forms have all of the serine/threonine residues in the N-terminus of Rfa2 mutated to aspartic acids (*rfa2-D*_*x*_) to mimic hyper-phosphorylation or alanines (*rfa2-A*_*x*_) to prevent phosphorylation. The phenotypes of these two mutant forms of *rfa2* are as follows: *rfa2-D*_*x*_ displays sensitivity to DNA damaging agents, whereas *rfa2-A*_*x*_ cells are resistant to DNA damage similar to WT *RFA2* cells (S2 Fig.). Interactions were then assessed in these strains to determine if the N-terminus might play a role in regulating the Rfa1-Rad24 interaction. There was no discernible lack of growth observed on SG-HTUL (Fig. 2E), regardless of the *RFA2* strain background in which the interaction was examined. This would indicate that the state of the Rfa2 N-terminus does not play an obvious role in regulating the Rfa1-Rad24 interaction in yeast cells.

### Putative phosphorylation of the Rad24 C-terminus is not regulating the Rfa1-Rad24 interaction

**Fig 3:**
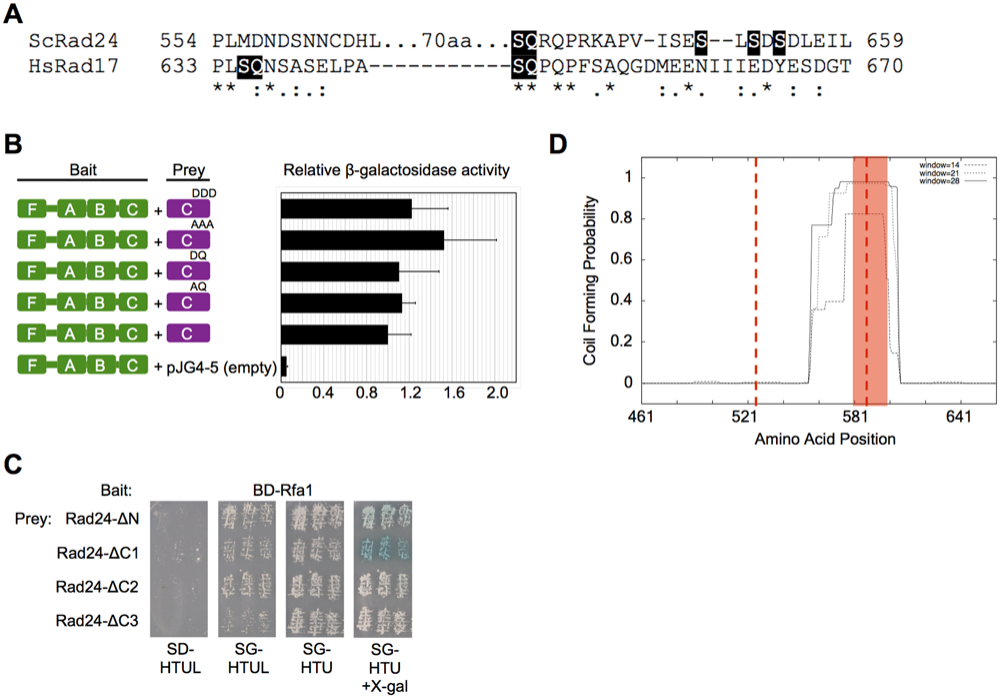
Determining the region of the Rad24 C-terminus required for Rfa1-Rad24 interaction. [A] T-COFFEE alignment of the C-termini of yeast Rad24 and the human homolog Rad17. Residues (S635 and S645) known to be phosphorylated [51] in human Rad17 (HsRad17) and their corresponding SQ motifs are highlighted. Residues (S650, S652, and S654) that have been reported by mass spectrometry to be phospho-targets [52,53] are highlighted for Rad24. In addition, a putative phosphorylation site (S637) is also denoted for Rad24 by highlighting the SQ motif. Asterisks indicate sequence identity, colons and periods indicate strongly and weakly similar residues, respectively. Rad24 contains an additional 70 aa region, which is denoted. [B] Phospho-mutant forms of the Rad24 C-terminus do not affect interaction. Interactions between Rfa1 and the C-terminal peptide of Rad24 containing mutations of putative phosphorylation sites were measured by liquid β-galactosidase assays. Values were normalized as in Fig. 2A. The mutant form of the Rad24 C-terminus is shown above each schematic. [C] Mapping the Rfa1 interaction region on Rad24. The C-terminus was divided into three subregions (~66 aa per subregion), and each subregion was deleted (ΔC1, ΔC2, ΔC3). Interaction with each subregion deletion peptide was examined by replica plating as described in Fig. 1A. [D] Secondary structure prediction of the Rad24 C-terminus. Jpred3 and COILS reveal a high probability for the formation of a coiled structure that overlaps subregions 2 and 3. The graph represents the COILS output file, and the red shading is the region predicted by Jpred3 to contain a coiled domain. Vertical red dashed lines denote the boundaries of the three subregions.

It is clear that treatment of cells with DNA damaging agents leads to disruption of the Rfa1-Rad24 two-hybrid interaction. However, this disruption is not easily explained by the potential post-translational modification of the Rfa2 N-terminus, based on the observation that the Rfa2 N-terminus phospho-mimetic mutant (*rfa2-D*_*x*_) has little effect on the Rfa1-Rad24 interaction in the context of an RFA complex (Fig. 2E). A plausible explanation is that Rad24 (specifically the C-terminus), not Rfa2, is post-translationally modified in response to DNA damage, resulting in the observed disruption of interaction with Rfa1. Because post-translational modification, particularly phosphorylation, of Rad24 is not well characterized, and phosphorylation of this region in human Rad17 occurs in response to DNA damage [50,51], we utilized PhosphoGRID (http://www.phosphogrid.org) to identify putative phosphorylation sites of Rad24 (Fig. 3A). This search revealed three sites that were identified through global mass spectrometry screens. These sites were serines located at amino acid positions 650, 652, and 654 of the 659 aa protein [52,53]. We also examined the importance of another serine located at amino acid position 637, as this is an SQ motif that is in a similar position to one of two SQ motifs (S635 and S645) in human Rad17 that have been demonstrated to be phosphorylated in response to DNA damage (Fig. 3A) [51]. Rad17 phosphorylation *in vitro* also requires that it is in the context of Rad17-RFC, and that ssDNA, human RPA, TopBP1, and ATR are present [17]. An alanine/aspartic acid mutagenesis approach was used to determine the importance of these serines for protein interaction with Rfa1. Fig. 3B reveals that β-galactosidase activity was not significantly altered, regardless of whether the Rad24 C-terminal fragment was mutated to phosphorylation mimetic (aspartic acid) forms or non-phosphorylatable (alanine) forms, indicating that none of these mutations led to significant disruption of interaction in the absence of DNA damage. This would indicate that post-translational modification of these residues is not accounting for the observed reduction in interaction between Rfa1 and Rad24 in DNA-damaged cells.

### Examining the role of a predicted coiled region and “conserved” RPA interaction motif for Rfa1-Rad24 interaction

The yeast two-hybrid screen allowed us to not only isolate candidates, known and novel, that interact with Rfa1, but also to narrow down the interaction to a specific region of the protein due to the fact that the genomic library did not contain whole genes, but gene fragments. As mentioned previously, the Rfa1-Rad24 interaction was narrowed down to the C-terminal 198 amino acids. Since there is no known structure for Rad24, we arbitrarily divided this C-terminal region into three subregions (~66 aa) to narrow this region further (Fig. 3C). We constructed a set of gene deletions that removed each of the three subregions individually. These deletion constructs were then examined for their ability to interact with Rfa1 by two-hybrid analysis. It was determined that deleting the first third (aa 461-527; Rad24-ΔC1) did not disrupt the interaction (Fig. 3C). However, deletion of either aa 528-594 (Rad24-ΔC2) or aa 595-659 (Rad24-ΔC3) resulted in disruption of the interaction as observed by a reduction in beta-galactosidase activity (Fig. 3C; white patches on SG-HTU+X-gal). Furthermore, less growth is observed for BD-Rad24-ΔC3 on SG-HTUL, indicating stronger disruption of the interaction. This implies that either the interaction region overlaps both missing regions, or that deleting one subregion disrupts structure of the adjacent subregion. The interaction was affected similarly whether or not Rfa1 was in the context of a complete RFA complex (see Rfa1-FLAB; S3 Fig.).

To determine if there is any potential structure in these subregions, we utilized two secondary structure prediction programs. The program Jpred3 (http://www.compbio.dundee.ac.uk/www-jpred/) predicted that the C-terminal region of Rad24 contains a coil from aa 581-601. The program Coils (http://embnet.vital it.ch/software/COILS_form.html) corroborated this prediction by also predicting a coil in the region of aa 568-604 (Fig. 3D). This predicted coil overlaps the subregions deleted above that resulted in reduced protein interaction. We also utilized these programs to examine human Rad17, the homolog of yeast Rad24. However, human Rad17 does not contain an obvious coil domain in the C-terminal region, and the predicted coil of Rad24 is located in the least conserved region between the two proteins, specifically within the ~70 additional amino acids found only in the yeast Rad24 C-terminus (Fig. 3A).

### Examination of a putative Rfa1 N-terminal interaction motif found in the predicted coil of Rad24

**Fig 4:**
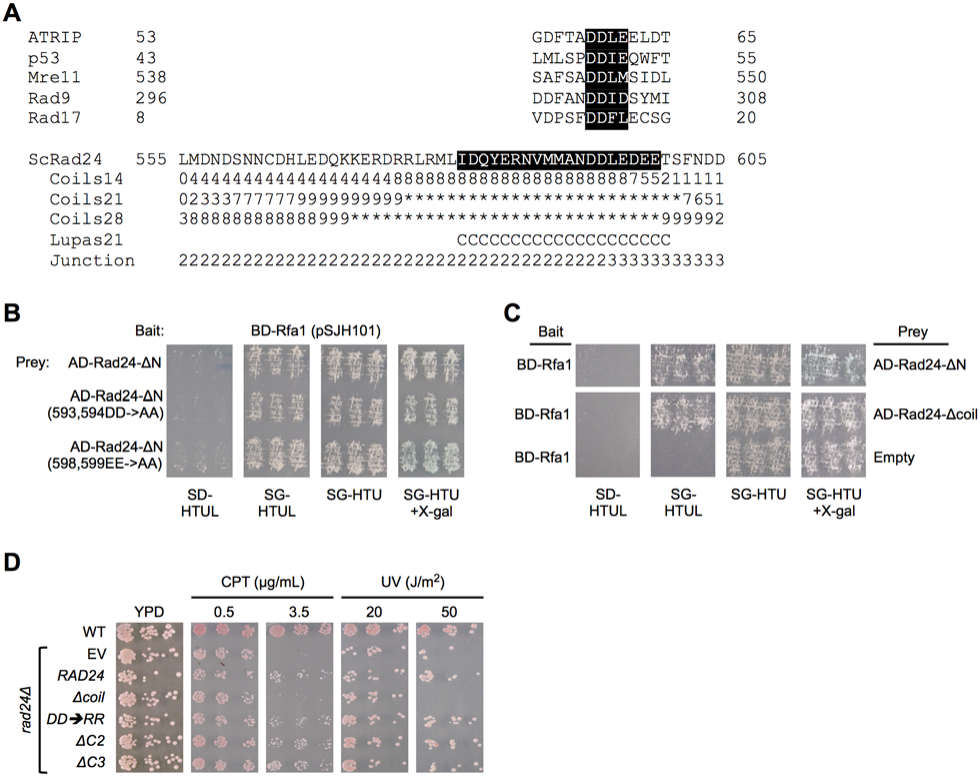
Examination of a putative interaction motif and the predicted coiled region of Rad24 and their role in the Rfa1-Rad24 interaction and cellular response to DNA damage. [A] Sequence display of Rad24 C-terminal region and the residues predicted to form a coiled structure. Jpred3 prediction probabilities are denoted below each residue, where the single digit represents the probability on a scale of 0-10 (*e*.*g*., 9 = 90%), rounded to the nearest digit. Probability approaching 100% is denoted by an asterisk. COILS examined the region in windows of 14, 21, and 28 amino acid residues (denoted for each). The denoted coil (C) from Jpred3 was predicted using Lupas [39] and was only predicted for a window of 21 aa. The predicted coil region is denoted by black highlighting. Junction represents the region that was deleted in ΔC2 (2) or ΔC3 (3). Above the amino acid sequence for Rad24 are “interaction motifs” predicted by Oakley and Patrick based on known interaction regions for these human proteins and human Rpa1 [1]. [B] Mutation of a predicted Rfa1 interaction motif does not disrupt interaction. The aspartic acid residues (D593 and D594) in the motif DDLE were mutated to alanines in the Rad24 C-terminal peptide, and interaction with Rfa1 was assessed via two-hybrid analysis as in Fig. 1C. Also examined was a mutant form where two glutamic acid residues (E598 and E599) in the predicted coil were mutated to alanines. Both mutant forms display growth on SG-HTUL and blue color on SG-HTU+X-gal that is as robust as the Rfa1-Rad24-ΔN interaction. [C] Deletion of the coil partially disrupts interaction. The predicted coil region was deleted and interaction between Rfa1 and the Rad24 C-terminus was assessed by replica plating as in [B]. Blue color on SG-HTU+X-gal is reduced compared to that for Rfa1-Rad24-ΔN. [D] Physiological consequences of deleting or mutating the Rad24 C-terminal region. DNA damage spot assays were performed, and the entire range of testing is shown in S4 and S5 Figs. *rad24*Δ cells (denoted vertically on left) containing an empty vector (EV) are sensitive to camptothecin (CPT) and ultraviolet radiation (UV). This can be complemented by a plasmid expressing the wild-type *RAD24* gene from its endogenous promoter. The following mutations were made in this plasmid and examined for sensitivity to CPT or HU: *DD→RR* = charge reversal mutant for D593 and D594; Δ*coil* = deletion of amino acids (aa) 575–601 containing the predicted coil; Δ*C2* or Δ*C3* = deletions of aa 528–594 or 595–659, respectively. WT denotes an isogenic strain containing the wild-type copy of *RAD24* in the chromosome.

Sequences or peptides that interact with DBD-F of Rpa1 were compared by Patrick and Oakley [1,54], and a putative consensus motif that is important for proteins to interact with the Rpa1 N-terminus was proposed. Using the sites of interaction for p53, ATRIP, Mre11, and Rad9, the proposed consensus amino acid sequence was DD(L/I)(E/D/M) [1,54], where the first two negatively charged residues appear to be most important. This sequence motif was then used to predict sites of other known RPA interactors that might be important for the interaction. One of these predicted sites was located from amino acids 8-20 (the N-terminus) of human Rad17 (isoform 1), with aa 13-16 containing the sequence DDFL [1] (Fig. 4A). Secondary structural predictions have already shown that this is not a region where a coil is predicted for either the yeast or human protein. Also, this region is located in the N-terminus of Rad17, and although we cannot rule out that this region is important for interaction with human RPA with these studies, we can definitively say that our identified interaction lies in the C-terminal region of Rad24 (Figs. 2B, 3B, and 3C).

Interestingly, Rad24 does contain a motif that looks similar to that reported among proteins that interact with the Rpa1 N-terminus. This motif is DDLE, and is located at aa 593-596, which overlaps the junction between the two subregion deletions that show disrupted interactions (Fig. 4A). To assess if this is the site of interaction, we generated amino acid substitution mutants where two adjacent negatively charged amino acids were mutated to alanines either at this site (593,594DD→AA) or at a nearby region (598,599EE→AA), both of which lie in the predicted coil. Examination of these mutants revealed no apparent disruption of interaction (growth of SG-HTUL and light blue color on SG-HTU+X-gal) compared to the unaltered Rad24 C-terminal fragment (Fig. 4B).

It is possible that structure of the coil is what is important. To assess this, the predicted coil region was deleted (Rad24-Δcoil) and interaction with BD-Rfa1 was measured. Replica plating demonstrated reduced β-galactosidase activity (white on the SG-HTU+X-gal plate); however, growth still occurred on the SG-HTUL plate (Fig. 4C). This indicated a reduced, but not completely disrupted interaction. However, this reduced interaction is similar to that observed for Rfa1-t11 or Rad24-ΔC2 constructs.

Given that interactions between Rfa1 and the Rad24 C-terminus are reduced when subregion 2 (ΔC2), subregion 3 (ΔC3), or the predicted coil region (Δcoil) are deleted, it was important to address whether these mutations resulted in physiologically-significant phenotypes, particularly in response to DNA damage. To do this, these mutations were introduced into a *RAD24* gene expressed from its native promoter on a low-copy centromeric vector. The full-length *RAD24*-expressing vector complements the *rad24*Δ defect when cells are exposed to camptothecin (CPT) or ultraviolet (UV) radiation (Fig. 4D; row 3 compared to row 2). Deletions of amino acids (aa) 528–594 (*rad24-*Δ*C2*) or 595–659 (*rad24-*Δ*C3*) do not have a negative effect on Rad24 physiological function. It was shown in Fig. 4B that a mutation of aspartic acids in the predicted interaction motif to alanines at aa 593 and 594 of Rad24 did not disrupt interaction with Rfa1. To examine whether or not these residues might have a physiological role in Rad24 function, they were mutated to arginines (*rad24-DD→RR*; charge reversal) instead of alanines; however, Rad24 function was not compromised when cells contain this mutation (Fig. 4D and S5 Fig.). Finally, a *rad24* mutant encoding Rad24 with a deletion of aa 575–601 (*rad24-*Δ*coil*) showed a damage-sensitive phenotype indistinguishable from that of *rad24*Δ cells (Fig. 4D and S5 Fig.). Taken together, these data suggest that although Rfa1-Rad24 interaction is disrupted when the Rad24 C-terminus is deleted, there is not a significant physiological effect in the DNA damage response. It also appears that deleting the predicted coil region has a different effect than deleting subregion 2 or subregion 3. We cannot rule out that the deletion of the putative coil region may have significant effects on Rad24 protein structure; however, we can say that deletions of 66 aa (subregion 2 or 3) in the C-terminus have little effect on Rad24 function in response to DNA damage.

## Discussion

### The Rfa1-Rad24 interaction appears to be independent of interaction with Rfa2-3 or Rfc2-5

It is clear that RPA is necessary for Rad24-RFC recruitment, and our studies suggest a number of features of this recruitment. First, the interaction between Rfa1 and Rad24 is likely either a direct one, or at least does not require any of the other RFA or RFC subunits. This conclusion is based on the observation that the Rad24-Rfa1 interaction occurred in the absence of Rfa2, Rfa3, Rfc2, Rfc3, Rfc4, and Rfc5, as the original identification was identified between BD-Rfa1-FLAB and AD-Rad24 that only contained the C-terminal 198 amino acid residues. Furthermore, BD-Rfa1-DBD-F can also interact with the Rad24 C-terminal peptide. BD-Rfa1-FLAB does not interact with Rfa2 or Rfa3 (S1A Fig.), and we predict that neither does BD-Rfa1-DBD-F based on previous work [55,56].

**Fig 5:**
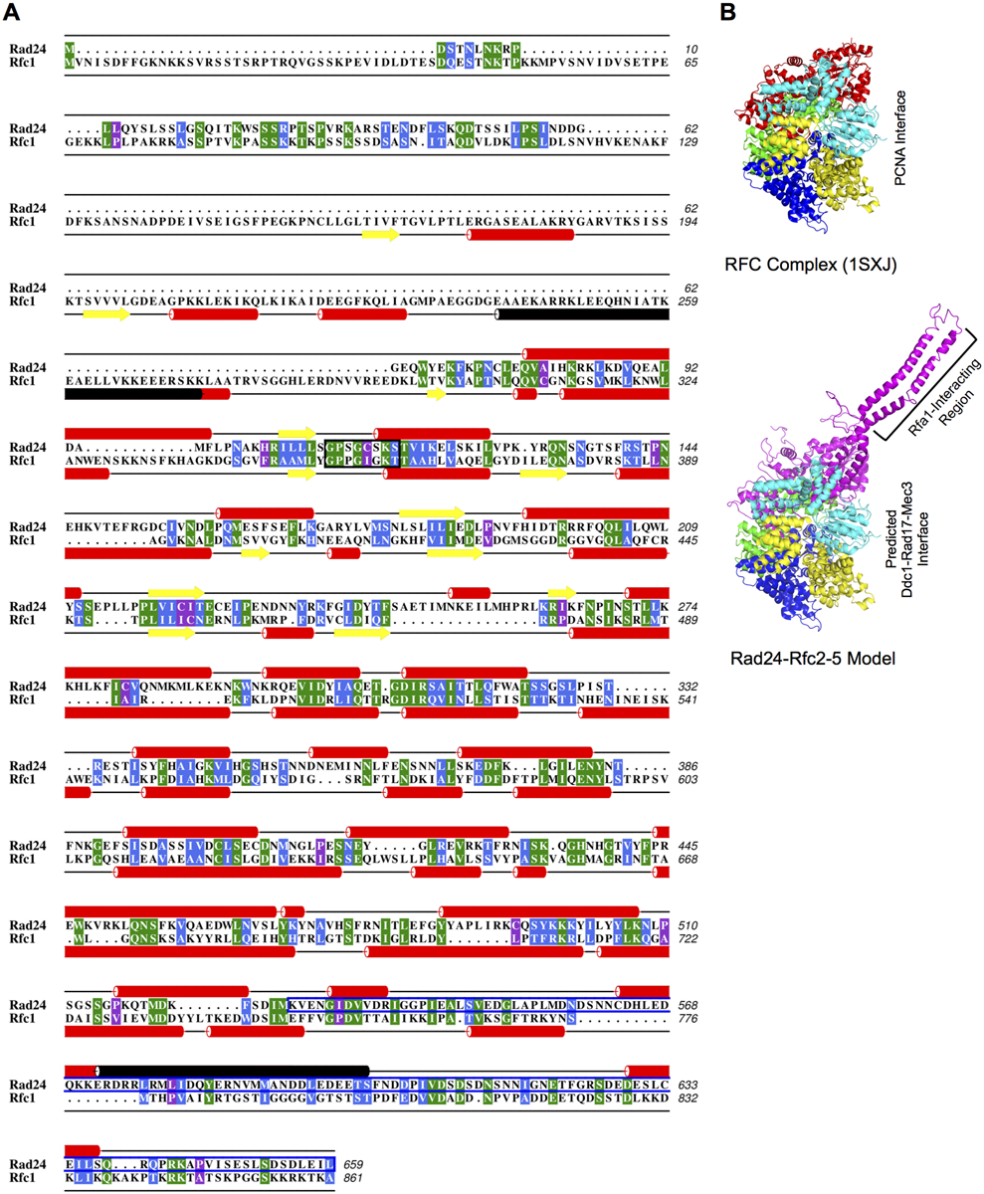
Sequence alignment and structural modeling support an Rfa1 interaction with Rad24-RFC that does not require Rfc2–5. [A] Consensus-based analysis of the Rad24 and Rfc1 protein sequence alignment and secondary structure predictions. Sequence alignment of Rad24 and Rfc1 from *Saccharomyces cerevisiae* was determined by T-COFEE [32]. The sequence alignment was edited using ALINE [40]. Sequence conservation is indicated as follows: green for invariant residues, blue for moderately conserved residues, and purple for weakly conserved residues. The NTP-binding motif [60] is outlined in black. The Rfa1-Rad24 interacting region (Rad24 aa 528–659) is outlined in blue. Secondary structure predictions were made using the online server PSIPred [61,62]. Alpha helical regions are indicated by red cylinders; beta strands are displayed using yellow arrows. The secondary structure prediction is displayed above and below the sequences for Rad24 and Rfc1, respectively. Black cylinders indicate sequences likely to adopt a coiled-coil conformation, as predicted by COILS/PCOILS [39,63] with residues of coiled-coil score distribution probabilities >50%. [B] Overview of the RFC complex incorporating a predicted Rad24 structure. A side-view of the crystal structure of the RFC complex (PDB ID: 1SXJ; left half) reveals a truncated Rfc1 protein in red (aa 295–785 of 861) in complex with Rfc5 (cyan), Rfc2 (yellow), Rfc3 (blue), Rfc4 (green), and ATP derivatives (not displayed) [57]. Structure predictions for Rad24 by Phyre2 [41], HHpred [42], I-TASSER [43], and RaptorX [44] each identified the core Rfc1 structure as a conserved structural motif shared by Rad24 (RaptorX: P-value score for this domain = 3.31e-07, where less than 10e-03 is considered a good model for mostly alpha helical proteins). Raptor X also modeled a C-terminal coiled-coil region in Rad24, corresponding to residues 473–652 (RaptorX: P-value score = 1.00e-02, indicating a moderate model). Although this region is greater than 35 residues, the estimated maximum length of a standard coiled-coil, this region overlaps with the PCOIL prediction (aa 572–601). When modeled in place of Rfc1 (red), the C-terminal region (denoted as Rfa1-Interacting Region) of Rad24 (magenta) lies outside of the 1SXJ crystal structure (right half). The sides representing the PCNA interface [57] and the predicted Ddc1-Rad17-Mec3 interface are denoted.

The region of Rad24 necessary for interaction mapped to the C-terminal 132 residues (aa 528-659). Based on a sequence alignment between Rad24 and Rfc1 (Fig. 5A), the Rad24 C-terminus is a good candidate for a regulatory region as it lies outside of the region identified as necessary for Rfc1 crystallization with Rfc2-5 as an RFC complex [57]. This would seem to preclude an interaction with another RFC subunit as necessary for the interaction between Rfa1 N-terminus and the Rad24 C-terminus to occur. This does not preclude other RFC subunits from contributing to the stability of an RFA-Rad24-RFC interaction, as a previous study has demonstrated genetic and physical interactions between Rfa1 and Rfc4 [18]. These data merely suggest that interaction of Rfa1-Rad24 does not require Rfc4 (or other RFC subunits).

### The Rfa1-Rad24 interaction occurs in undamaged cells, is not DNA context dependent, and is disrupted upon DNA damage induction

Rad24-RFC is a clamp loader required to load the damage clamp Ddc1-Rad17-Mec3 onto damaged DNA templates and requires functional RFA for loading [15,16,49]. Therefore, it is of note that the Rfa1-Rad24 interaction observed occurs in unstressed cells, and that the interaction does not appear to be DNA context-dependent. This would imply that Rad24-RFC present in mitotically-growing cells potentially could be associated with a population of RFA prior to association with DNA, and that RFA might load Rad24-RFC as it binds to damaged DNA templates. This remains an open question, as previous demonstrations of damage clamp loader (yeast Rad24-RFC or human Rad17-RFC) loading always involved pre-incubation of DNA templates with RFA (or human RPA) before addition of Rad24-RFC (or human Rad17-RFC) onto a ssDNA-dsDNA junction [15,17].

One feature that was readily apparent with the Rfa1-Rad24 interaction was upon induction of DNA damage, the interaction, as measured by yeast two-hybrid assay was abrogated. This strongly indicates that the interaction is dependent on the DNA damage state of the cell. We propose three possibilities to explain this phenomenon. One possibility is that once Rad24-RFC is loaded onto the damaged template, the interaction between Rfa1 and Rad24 must be abolished to facilitate downstream loading of Ddc1-Rad17-Mec3 and/or release of RFA from the substrate in order for the substrate to be processed properly. Thus far, *in vitro* studies have only investigated the loading of the clamp loader and damage clamp. This is supported by a structural modeling of Rad24-RFC (Fig. 5B), in which the Rad24 C-terminus appears to extend towards the interface of RFC that would normally interact with PCNA [57]. If this same interface is necessary for Ddc1-Rad17-Mec3 loading, then continued RFA interaction might sterically inhibit its loading. Since RFA would be interacting with the ssDNA at or near the junction, this would potentially necessitate RFA removal (or at least Rad24-RFC sliding, requiring release from RFA) before Ddc1-Rad17-Mec3 could be loaded. A second, and not mutually-exclusive, possibility is a handoff mechanism in which upon DNA damage and loading of Rad24-RFC, Rad24 interaction with the N-terminus of RFA is actively replaced by yeast Ddc1 (human Rad9) of the damage clamp. Support for this possibility lies in the similarity in amino acid sequence of putative interaction motifs proposed for human Rad9 and yeast Rad24. In fact, it has been suggested that a number of factors required for the DNA damage response interact with the Rfa1 N-terminus, and that these interactions may occur through similar motifs (*e*.*g*., DDLE) and at similar locations with Rfa1 (*e*.*g*., basic cleft of DBD-F in the Rpa1 N-terminus). It is difficult to imagine how these factors could simultaneously interact due to steric hindrance, unless Rfa1 uses a “catch and release” mechanism to coordinate association with these factors. This would most likely require post-translational modification of RFA and/or the associating factor. We have demonstrated that mimicking post-translational modification of the Rfa2 N-terminus or Rad24 C-terminus does not have an apparent effect on Rfa1-Rad24 interaction; however, it has been shown *in vitro* that a human Rpa2 phospho-mimetic is less efficient at loading Rad17-RFC and displays a decrease in phosphorylation of Rad17 by ATR [17]. As this assay is performed in yeast cells exposed to a DNA damaging agent, it is possible that other factors might be regulating the Rfa1-Rad24 interaction. A third possibility is that upon DNA damage, the Rfa1 and AD-Rad24 interaction actually gets stronger. In this alternative scenario, AD-Rad24 is recruited away from the lexA operator sequence and onto the damaged DNA substrate bound by either BD-Rfa1 or endogenous Rfa1, resulting in a reduction in reporter gene expression and the appearance of a disrupted interaction. Finally, we cannot rule out that the Rad24 C-terminal fragment might have reduced stability when cells are treated with MMS. These possibilities cannot be distinguished easily using the two-hybrid assay.

### Interaction with Rad24 and loading of Rad24-RFC may be separable functions of RFA

One observation was that a form of Rfa1 protein that contained a charge-reversal mutation (K45E; Rfa1-t11) still interacted with the Rad24 C-terminus, at least above background. This was supported by the appearance of growth on SG-HTUL plates, a very faint blue color observed on SG-HTU+X-gal plates, and by quantitative β-galactosidase measurement. Our data are consistent with deficient loading Rad24-RFC onto a damaged DNA template by Rfa1-ΔN (DBD-F removal) being due to lack of interaction with Rad24-RFC [15]. However, given that Rfa1-t11 shows interaction with Rad24, we propose that the lack of Rad24-RFC loading by Rfa1-t11 could also be explained by either reduction of interaction with another RFC subunit (Rfc4; [18]) or an inability of Rfa1-t11 to recognize and/or interact with the damaged DNA template properly. It is clear that yeast RFA (or human RPA) containing the t11 or ΔN mutation bind these templates; it is not clear that they are bound in the exact same fashion as WT RFA. We acknowledge this is quite difficult to determine; however, it should not be dismissed as a possibility. It also does remain possible that the interaction between Rfa1-t11 and Rad24 might be below the threshold necessary to load Rad24-RFC.

### Is there an interaction motif for proteins that interact with DBD-F?

The fact that the few proteins where interaction has actually been mapped to a specific region or specific amino acids contain a sequence that might be important for electrostatic interaction with the basic cleft of DBD-F is promising for easier identification of an interaction motif in other proteins. Oakley and Patrick indicated this by making the prediction for where this interaction region is located for a few other RPA interactors, even indicating where this sequence might be located in a phosphorylated Rpa2 (EpSYG, where pS is phosphorylated serine at amino acid position 8), although it has not yet been tested if phosphorylation of serine 8 in Rpa2 is the residue that is directly responsible for disrupting known protein interactions [48] through steric inhibition. Mutation of the putative consensus and deletion of the predicted coil region of the Rad24 C-terminus suggest that the coil might have some importance, but does not support DDLE as the short consensus Rfa1 DBD-F binding motif, at least for Rad24. However, the C-terminus of Rad24 appears to be acidic (33/128 aspartic and glutamic acid residues *vs*. 13/128 lysine and arginine residues). This might explain why the Rad24 C-terminus interacts with DBD-F of Rfa1 (perhaps through its basic cleft, similar to other DBD-F interactors). However, it does not explain why this domain does not interact with DBD-A, DBD-B, or DBD-C, all of which have basic cleft regions. This would suggest that there is an additional structural feature or sequence necessary for interaction with DBD-F specifically.

### The loading of Rad24-RFC mediated through Rfa1 may also be mediated through the other RFC/RFA proteins

Although four mutations (*rfa1-t11*, *rad24-*Δ*coil*, *rad24-*Δ*C2*, and *rad24-*Δ*C3*) examined in this study appear to dramatically reduce Rfa1-Rad24 interaction, two of these have no observable damage-sensitivity phenotype. *rad24-*Δ*C2* and *rad24-*Δ*C3* (which appeared to have the greatest reduction in interaction) mutant cells are indistinguishable from WT *RAD24* cells when treated with multiple DNA damaging agents. Also, *rad24-*Δ*coil* mutant cells display observable sensitivities to camptothecin (CPT) or ultraviolet (UV) light, similar to *rad24*Δcells, whereas *rfa1-t11* cells display sensitivities to CPT and UV that are more severe [58]. Therefore, we cannot definitively correlate the reduction of interaction between Rfa1-Rad24 with the damage-sensitivity phenotypes observed and presumably loading of Rad24-RFC. We propose that the interaction between the Rfa1 N-terminus and the Rad24 C-terminus may not be the exclusive driving force for Rad24-RFC loading. Rather, other RFC (and potentially other RFA) subunits are likely involved. Evidence supporting this lies in the interaction identified between the Rfa1 N-terminus and Rfc4, which is also disrupted by Rfa1-t11 (and other mutations that lie in the Rfa1 N-terminus) [18]. Also supporting this is the apparent ability for Rad24-RFC to function when the Rfa1-Rad24 interaction should be disrupted. Since RFA is absolutely necessary for loading Rad24-RFC, one must presume that Rad24 and/or other RFC subunits still can interact with Rfa2, Rfa3, or other regions of Rfa1 to facilitate loading in the absence of the identified Rfa1-Rad24 interaction; otherwise, a damage-sensitive phenotype should have been observed for *rad24-*Δ*C2* or *rad24-*Δ*C3* cells.

Using a combination of yeast two-hybrid analysis and mutagenesis, we determined that the Rfa1-interacting region of Rad24 is in the C-terminus. This screen also identified other previously characterized interactors (*e*.*g*., Sgs1 [8]; Mph1 [59]; Dna2 [9]), including their region of interaction. These interactors have also been indicated to require the Rfa1 N-terminus for interaction. The fact that these interactions all happen at the N-terminus of Rfa1 indicates one of two possibilities: the interactions are either temporally distinct or observed as part of a mega-complex of proteins in which one or a few interact with DBD-F. The fact that the two-hybrid constructs we used and identified for Rfa1 and Rad24 lacked regions important for interactions with Rfa2-Rfa3 and Rfc2-5, respectively, also lends support to a more direct interaction. Once more interactors have been carefully examined and mapped to the N-terminus of Rfa1, it will be important to determine whether interactions are sequential (temporal) or spatial, what additional factors are necessary, and the condition-specificity for interactions.

## Supporting Information

S1 Fig Characterization of additional bait constructs examined. [A] Complementation analysis of Rfa1 bait constructs. RMY122-A (*rfa1*Δ::*TRP1 rfa2*Δ::*TRP1*) cells were co-transformed with one of the bait plasmids (Table 2) and pAW07 (*RFA2*). Plasmid shuffle was used to determine if cells could lose pJM132 (*RFA1* and *RFA2*) and grow on 5-FOA-containing media. SD-HLU = growth control. Lack of growth on SD+5-FOA indicates lack of viability and lack of RFA function. A negative (-) control is shown that contains another truncated BD-Rfa1, and Rfa1-BD represents Rfa1 containing a C-terminal lexA tag (despite partial complementation of *rfa1*Δ, this construct does not display interaction with AD-Rfa2 or AD-Rfa3 and was not used further). Expression of all bait proteins is driven by the *ADH1* promoter, except for pPM07 (driven by *GAL1* promoter; expression is leaky on SD+5-FOA).[B] Interactions with Rfa2 and Rfa3. EGY48 (6x*O*_*lexA*_-*LEU2*) cells were co-transformed with pPM07 (BD-Rfa1) or pENM17 (BD-Rfa1) and pENM10 (B42-Rfa1), pENM11 (B42-Rfa2), or pENM12 (B42-Rfa3) and pSH18-34 (8x*O*_*lexA*_-*lacZ*). Nine independent transformants (three are shown) were assayed as described in Fig. 1A.[C] Characterization of the interaction between Rfa1-FLAB and Rad24 or Sgs1. The prey plasmids pGP2, encoding a Rad24 peptide (Rad24-ΔN) containing the C-terminal 198 amino acids (residues denoted in parentheses) or pGP3, encoding an Sgs1 peptide containing central amino acids 421-792 (denoted in parentheses), were co-transformed with pGP1 (BD-Rfa1-FLAB) and pSH18-34. Three independent colonies (two shown) were examined as described in Fig. 1C.
(TIF)

S2 Fig DNA damage phenotypes of *rfa2* N-terminal phospho-mimetic and non-phosphorylatable mutants. EGY48 derivatives containing an *RFA2* gene where all 10 serines/threonines within the first 34 aa were mutated to aspartic acids (*rfa2-D*_*x*_; mimic phosphorylated state) or alanines (*rfa2-A*_*x*_; prevent phosphorylation). Cellular resistance to DNA damaging agents was examined by spotting serial dilutions of cells onto media containing DNA damaging agents. Although reported elsewhere for the RMY122-A and JKM179 strain backgrounds [58], these mutations display the same phenotypes in the EGY48 background: *rfa2-D*_*x*_ displays damage-sensitivity and *rfa2-A*_*x*_ displays damage-resistance.
(TIF)

S3 Fig Mapping the Rfa1-FLAB interaction region on Rad24. Assay was performed as in Fig. 3C, except BD-Rfa1-FLAB was used as the bait. This demonstrates that interaction is not mediated by Rfa2 or Rfa3.
(TIF)

S4 Fig Verifying complementation of a *RAD24*-expressing plasmid with its endogenous promoter and determination of damaging agents that cause sensitivity in a *rad24Δ* strain. A *RAD24*-expressing plasmid was constructed by cloning in the *RAD24* gene and either 500 base pairs (bp) (*P*_*500*_) or 1,000 bp (*P*_*1*,*000*_) of endogenous upstream sequence. These were tested for complementation under various types and concentrations of DNA damaging agents as described in Fig. 4D. Sensitivity was only observed for *rad24*Δ cells when treated with ultraviolet radiation or camptothecin, and all of the *RAD24* constructs show complementation (two independent plasmids for each).
(TIF)

S5 Fig Examining physiological relevance of *rad24* C-terminal mutants. *rad24* mutant constructs tested were as described in Fig. 4D. The full range of testing is shown here. Select plates were used for Fig. 4D.
(TIF)

S1 Table Yeast strains. (DOCX)

S2 Table Plasmids. (DOCX)

S3 Table Primers. (DOCX)

S4 Table Other novel putative Rfa1-FLAB interactors. (DOCX)
