# Supplementary figures and images for: Characterization of the Interaction between Rfa1 and Rad24 in Saccharomyces cerevisiae
Source: PLoS One. 2015 Feb 26;10(2):e0116512. doi: 10.1371/journal.pone.0116512 (PMC4342240; doi:10.1371/journal.pone.0116512)

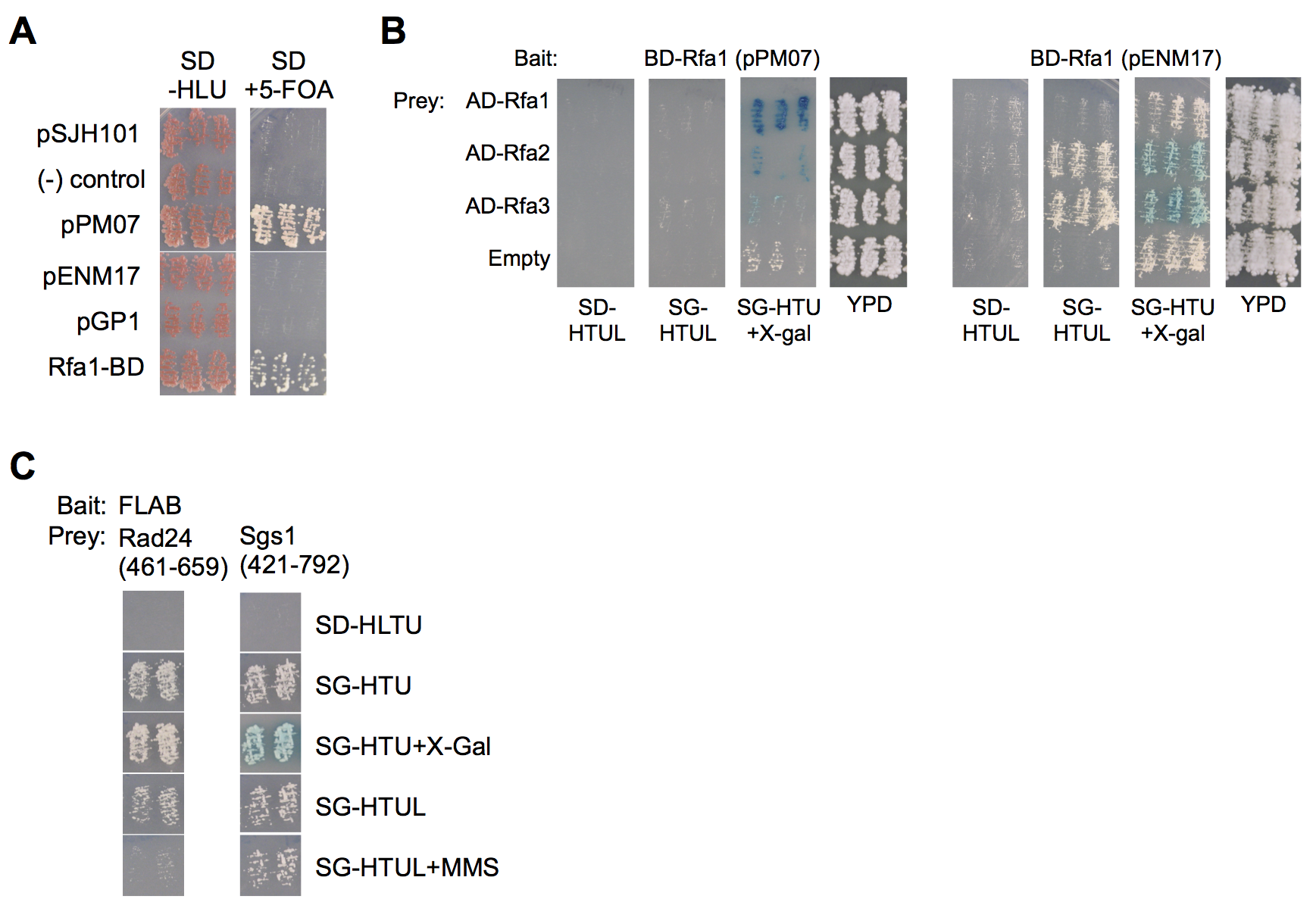

Supplement: S1 Fig — [A] Complementation analysis of Rfa1 bait constructs. RMY122-A (rfa1Δ::TRP1 rfa2Δ::TRP1) cells were co-transformed with one of the bait plasmids (Table 2) and pAW07 (RFA2). Plasmid shuffle was used to determine if cells could lose pJM132 (RFA1 and RFA2) and grow on 5-FOA-containing media. SD-HLU = growth control. Lack of growth on SD+5-FOA indicates lack of viability and lack of RFA function. A negative (-) control is shown that contains another truncated BD-Rfa1, and Rfa1-BD represents Rfa1 containing a C-terminal lexA tag (despite partial complementation of rfa1Δ, this construct does not display interaction with AD-Rfa2 or AD-Rfa3 and was not used further). Expression of all bait proteins is driven by the ADH1 promoter, except for pPM07 (driven by GAL1 promoter; expression is leaky on SD+5-FOA).[B] Interactions with Rfa2 and Rfa3. EGY48 (6xO lexA-LEU2) cells were co-transformed with pPM07 (BD-Rfa1) or pENM17 (BD-Rfa1) and pENM10 (B42-Rfa1), pENM11 (B42-Rfa2), or pENM12 (B42-Rfa3) and pSH18-34 (8xO lexA-lacZ). Nine independent transformants (three are shown) were assayed as described in Fig. 1A.[C] Characterization of the interaction between Rfa1-FLAB and Rad24 or Sgs1. The prey plasmids pGP2, encoding a Rad24 peptide (Rad24-ΔN) containing the C-terminal 198 amino acids (residues denoted in parentheses) or pGP3, encoding an Sgs1 peptide containing central amino acids 421-792 (denoted in parentheses), were co-transformed with pGP1 (BD-Rfa1-FLAB) and pSH18-34. Three independent colonies (two shown) were examined as described in Fig. 1C. (TIF) [file pone.0116512.s001.tif]

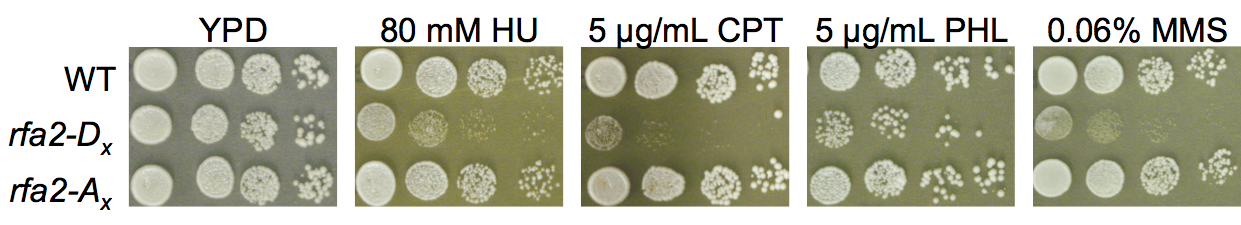

Supplement: S2 Fig — EGY48 derivatives containing an RFA2 gene where all 10 serines/threonines within the first 34 aa were mutated to aspartic acids (rfa2-D x; mimic phosphorylated state) or alanines (rfa2-A x; prevent phosphorylation). Cellular resistance to DNA damaging agents was examined by spotting serial dilutions of cells onto media containing DNA damaging agents. Although reported elsewhere for the RMY122-A and JKM179 strain backgrounds [58], these mutations display the same phenotypes in the EGY48 background: rfa2-D x displays damage-sensitivity and rfa2-A x displays damage-resistance. (TIF) [file pone.0116512.s002.tif]

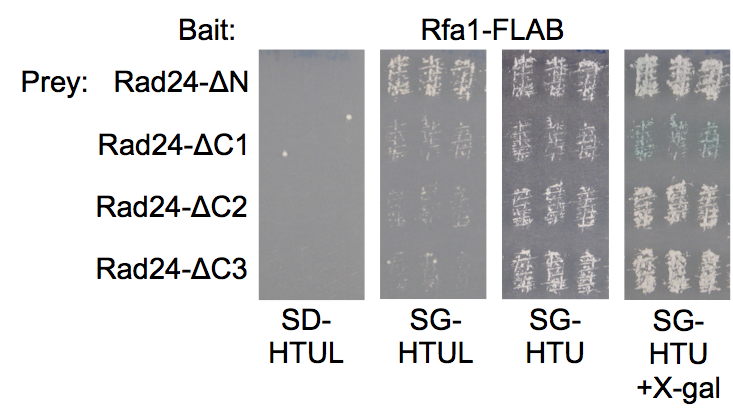

Supplement: S3 Fig — Assay was performed as in Fig. 3C, except BD-Rfa1-FLAB was used as the bait. This demonstrates that interaction is not mediated by Rfa2 or Rfa3. (TIF) [file pone.0116512.s003.tif]

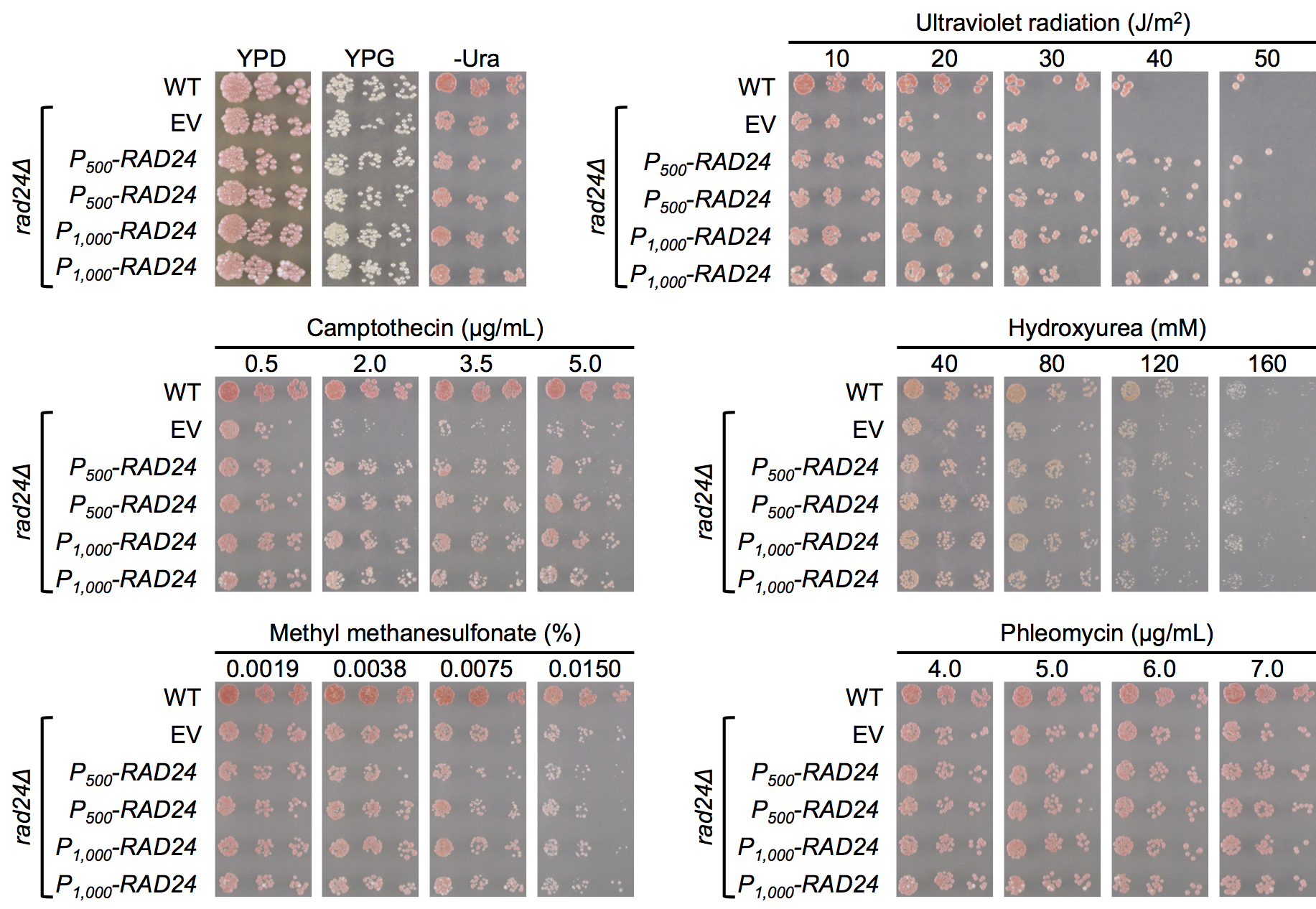

Supplement: S4 Fig — A RAD24-expressing plasmid was constructed by cloning in the RAD24 gene and either 500 base pairs (bp) (P 500) or 1,000 bp (P 1,000) of endogenous upstream sequence. These were tested for complementation under various types and concentrations of DNA damaging agents as described in Fig. 4D. Sensitivity was only observed for rad24Δ cells when treated with ultraviolet radiation or camptothecin, and all of the RAD24 constructs show complementation (two independent plasmids for each). (TIF) [file pone.0116512.s004.tif]

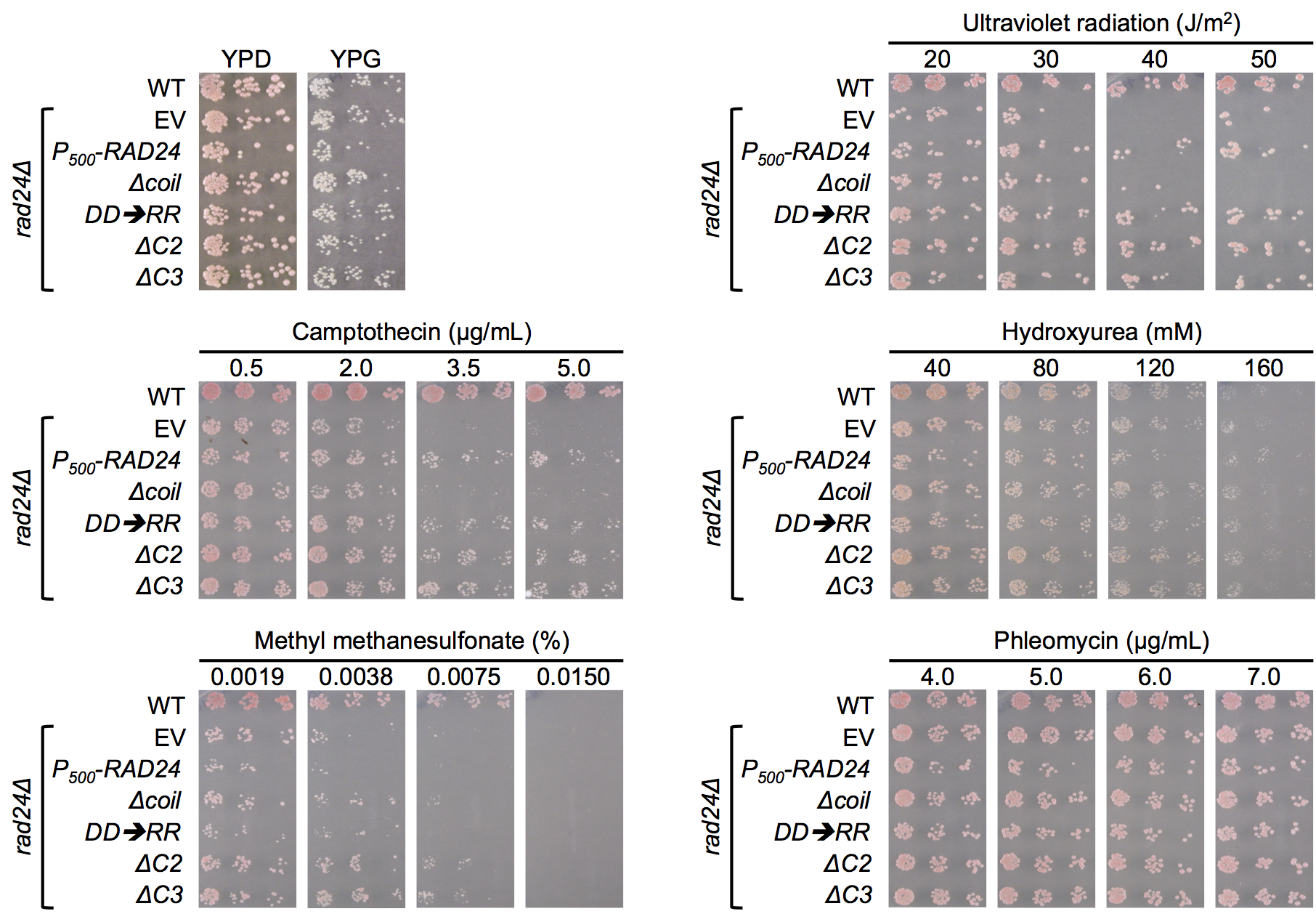

Supplement: S5 Fig — rad24 mutant constructs tested were as described in Fig. 4D. The full range of testing is shown here. Select plates were used for Fig. 4D. (TIF) [file pone.0116512.s005.tif]
