# Supplementary material for: Characterization of the Interaction between Rfa1 and Rad24 in Saccharomyces cerevisiae
Source: PLoS One. 2015 Feb 26;10(2):e0116512. doi: 10.1371/journal.pone.0116512 (PMC4342240; doi:10.1371/journal.pone.0116512)
Supplement: S1 Table — (DOCX) [file pone.0116512.s006.docx]

**Table S1. Yeast strains**

| Strain Name | Genotype/Description | Reference |
| --- | --- | --- |
| EGY48 | *MAT‑α his3 trp1 ura3 leu2::6xO_lexA_‑LEU2* | [29] |
| EGY188 | *MAT‑a his3 trp1 ura3 leu2::2xO_lexA_‑LEU2* | [29] |
| EGY48‑rfa2‑D_x_ | *MAT‑α his3 trp1 ura3 leu2::6xO_lexA_‑LEU2 rfa2‑D_x_* | This study |
| EGY48‑rfa2‑A_x_ | *MAT‑α his3 trp1 ura3 leu2::6xO_lexA_‑LEU2 rfa2‑A_x_* | This study |
| EGY48‑rfa2‑ΔN_x_ | *MAT‑α his3 trp1 ura3 leu2::6xO_lexA_‑LEU2 rfa2‑ΔN_x_* | This study |
| RMY122‑α | *MAT‑α his3‑11,15 leu2‑3,112 ura3‑1 trp1‑1 ade2‑1 can1‑100 rad5‑G535R rfa1Δ::TRP1 rfa2Δ::TRP1*; contains plasmid pJM132 | [27] |
| RMY122‑A | *MAT‑a* derivative of RMY122‑α; contains plasmid pJM132 | This study |
| RMY122-A-rad24Δ | *MAT‑a* derivative of RMY122‑A containing *rad24Δ::kanMX*; contains plasmids pRS313-RFA1 and pRS315-RFA2 | This study |
