# Supplementary material for: Characterization of the Interaction between Rfa1 and Rad24 in Saccharomyces cerevisiae
Source: PLoS One. 2015 Feb 26;10(2):e0116512. doi: 10.1371/journal.pone.0116512 (PMC4342240; doi:10.1371/journal.pone.0116512)
Supplement: S2 Table — (DOCX) [file pone.0116512.s007.docx]

**Table S2. Plasmids**

| Name | Genotype/Description | Reference |
| --- | --- | --- |
| pJM132 | pRS416 containing *RFA1*, *RFA2* cDNA, and *RFA3*; *URA3*; *amp^r^* | [27] |
| pRS313‑RFA1 | pRS313 containing *RFA1* expressed from its native promoter; *HIS3*; *amp^r^* | This study |
| pAW07 | pRS315 containing *RFA2* expressed from its native promoter; *LEU2*; *amp^r^* | This study |
| pEG202 | Bait vector; lexA (BD) fusion protein expression driven by constitutive promoter (*P_ADH1_*); *HIS3*; 2 μ; *amp^r^* | [28,29] |
| pEG202K | Derivative of pEG202 containing *amp^r^Δ::kanMX* (*i.e.*, *kan^r^*) | This study |
| pGP1 | pEG202 derivative expressing lexA‑rfa1‑FLAB fusion protein; Rfa1 is truncated (missing DBD‑C) due to nonsense mutation (Trp→STOP) at amino acid (aa) 413 | This study |
| pJG4‑5 | Prey vector; B42 (AD)‑HA fusion protein expression driven by inducible promoter (*P_GAL1_*); *TRP1*; 2 μ; *amp^r^* | [28,29] |
| pGP2 | pJG4‑5 derivative isolated from two‑hybrid screen expressing the C‑terminal region (aa 461‑659) of Rad24 (also referred to as *rad24‑ΔN*) | This study |
| pGP3 | pJG4‑5 derivative isolated from two‑hybrid screen expressing the central region (aa 421‑792) of Sgs1 | This study |
| pGP17 | pJG4‑5 derivative expressing the full‑length WT Rad24 generated by PCR and *in vivo* homologous recombination cloning | This study |
| pGP18 | pJG4‑5 derivative expressing the N‑terminal region (aa 1‑460) of Rad24 (also referred to as *rad24‑ΔC*) generated by PCR and *in vivo* homologous recombination cloning | This study |
| pSH18‑34 | Reporter vector containing *8xO_lexA_‑lacZ*; *URA3*; 2 μ; *amp^r^* | [29] |
| pGAL‑lexA | Bait vector; lexA (BD) fusion protein expression driven by inducible promoter (*P_GAL1_*) | [8] |
| pPM07 | pGAL‑lexA expressing lexA‑Rfa1 | [8] |
| pPM09 | pGAL‑lexA expressing lexA‑DBD‑F+Linker of Rfa1 | [8] |
| pPM11 | pGAL‑lexA expressing lexA‑DBD‑A of Rfa1 | [8] |
| pPM13 | pGAL‑lexA expressing lexA‑DBD‑B of Rfa1 | [8] |
| pPM15 | pGAL‑lexA expressing lexA‑DBD‑C of Rfa1 | [8] |
| p2069 | pGAL‑lexA expressing lexA‑DBD‑F of Rfa1 | [8] |
| p2068 | pGAL‑lexA expressing lexA‑Linker of Rfa1 | [8] |
| pSJH101 | pEG202K expressing lexA‑Rfa1 that was subcloned from pPM07; expression is driven from *P_ADH1_* instead of *P_GAL_* | This study |
| pTMW2 | pRS306‑like plasmid containing *rfa2‑D_x_*; *URA3*; *amp^r^* | This study |
| pTMW3 | pRS306‑like plasmid containing *rfa2‑A_x_*; *URA3*; *amp^r^* | This study |
| pTMW4 | pRS306‑like plasmid containing *rfa2‑ΔN_x_*; *URA3*; *amp^r^* | This study |
| pENM17 | pEG202 derivative expressing lexA‑Rfa1 (N‑terminal tag) fusion protein from the *ADH1* promoter | This study |
| pENM18 | pEG202 derivative expressing Rfa1‑lexA (C‑terminal tag) fusion protein from the *ADH1* promoter | This study |
| pENM10 | pJG4‑5 derivative expressing B42‑HA‑Rfa1 fusion protein; generated by PCR and *in vivo* homologous recombination cloning | This study |
| pENM11 | pJG4‑5 derivative expressing B42‑HA‑Rfa2 fusion protein; generated by PCR and *in vivo* homologous recombination cloning | This study |
| pENM12 | pJG4‑5 derivative expressing B42‑HA‑Rfa3 fusion protein; generated by PCR and *in vivo* homologous recombination cloning | This study |
| pENM6 | pGP2 derivative containing S650D, S652D, S654D mutations | This study |
| pENM7 | pGP2 derivative containing S650A, S652A, S654A mutations | This study |
| pENM8 | pGP2 derivative containing S637D mutation | This study |
| pENM9 | pGP2 derivative containing S637A mutation | This study |
| pGP19 | pGP2 derivative containing ΔC1 | This study |
| pGP20 | pGP2 derivative containing ΔC2 | This study |
| pGP21 | pGP2 derivative containing ΔC3 | This study |
| pENM20 | pGP2 derivative containing Δcoil | This study |
| pENM21 | pSJH101 containing *rfa1‑t11* | This study |
| pENM22 | pRS316 containing *RAD24* driven from its endogenous promoter | This study |
| pENM23 | pENM22 containing *rad24-DD593,594RR* mutation | This study |
| pENM24 | pENM22 containing *rad24-Δcoil* | This study |
| pENM25 | pENM22 containing *rad24-ΔC2* | This study |
| pENM26 | pENM22 containing *rad24-ΔC3* | This study |
