# Supplementary material for: Characterization of the Interaction between Rfa1 and Rad24 in Saccharomyces cerevisiae
Source: PLoS One. 2015 Feb 26;10(2):e0116512. doi: 10.1371/journal.pone.0116512 (PMC4342240; doi:10.1371/journal.pone.0116512)
Supplement: S3 Table — (DOCX) [file pone.0116512.s008.docx]

**Table S3. Primers**

| Primer # | Primer Name | Sequence (5’→3’) | Use |
| --- | --- | --- | --- |
| O‑338 | RFA2‑UP‑NEW | TAGCAATTCCTTTGGCCTCGATGAGCTTCC | Verify *rfa2* genomic mutation |
| O‑339 | RFA2‑DOWN‑NEW | GATAAAACCCTGGTCAGTCAAGGTCGTAC |  |
| O‑268 | pEG202‑RFA1‑FOR | TATTCGCAACGGCGACTGGCTGGAATTCCCGGGGATCCTTATGAGCAGTGTTCAACTTT | Cloning *RFA1* into pEG202 |
| O‑269 | pEG202‑RFA1‑REV | AAATTCGCCCGGAATTAGCTTGGCTGCAGGTCGACTCGAGTTAAGCTAACAAAGCCTTGG |  |
| O‑270 | pEG202‑RFA2‑FOR | TATTCGCAACGGCGACTGGCTGGAATTCCCGGGGATCCTTATGGCAACCTATCAACCATA | Cloning *RFA2* into pEG202 |
| O‑271 | pEG202‑RFA2‑REV | AAATTCGCCCGGAATTAGCTTGGCTGCAGGTCGACTCGAGTCATAGGGCAAAGAAGTTAT |  |
| O‑272 | pEG202‑RFA3‑FOR | TATTCGCAACGGCGACTGGCTGGAATTCCCGGGGATCCTTATGGCCAGCGAAACACCAAG | Cloning *RFA3* into pEG202 |
| O‑273 | pEG202‑RFA3‑REV | AAATTCGCCCGGAATTAGCTTGGCTGCAGGTCGACTCGAGCTAGTATATTTCTGGGTATT |  |
| O‑294 | pJG4‑5‑Rfa1‑FOR | CTACCCTTATGATGTGCCAGATTATGCCTCTCCCGAATTCATGAGCAGTGTTCAACTTT | Cloning *RFA1* into pJG4‑5 |
| O‑295 | pJG4‑5‑Rfa1‑REV | TTGACCAAACCTCTGGCGAAGAAGTCCAAAGCTTCTCGAGTTAAGCTAACAAAGCCTTGG |  |
| O‑296 | pJG4‑5‑Rfa2‑FOR | CTACCCTTATGATGTGCCAGATTATGCCTCTCCCGAATTCATGGCAACCTATCAACCATA | Cloning *RFA2* into pJG4‑5 |
| O‑297 | pJG4‑5‑Rfa2‑REV | TTGACCAAACCTCTGGCGAAGAAGTCCAAAGCTTCTCGAGTCATAGGGCAAAGAAGTTAT |  |
| O‑298 | pJG4‑5‑Rfa3‑FOR | CTACCCTTATGATGTGCCAGATTATGCCTCTCCCGAATTCATGGCCAGCGAAACACCAAG | Cloning *RFA3* into pJG4‑5 |
| O‑299 | pJG4‑5‑Rfa3‑REV | TTGACCAAACCTCTGGCGAAGAAGTCCAAAGCTTCTCGAGCTAGTATATTTCTGGGTATT |  |
| O‑340 | pJG4‑5‑UP‑Sequence | TGGCGGATCAGGCGATTAACGTGGTGCCGG | Sequence insert fusion junction at 5’ end |
| O‑391 | pJG4‑5‑RAD24‑FOR | ATGGATAGTACGAATTTGAA | Cloning *RAD24* into pJG4‑5 |
| O‑392 | pJG4‑5‑RAD24‑REV | TTAGAGTATTTCCAGATCTG |  |
| O‑468 | pJG4‑5‑rad24‑Delta(461‑659) | ATTCATTTAAAGTTCAAGCTTAACTCGAGAAGCTTTGGAC | Mutagenesis to generate *rad24‑ΔC* (deletion of aa 461‑659) |
| O‑415 | rad24‑C‑Term‑D | GCGCCAGTTATCAGTGAGGATCTTGATGATGATGATCTGGAAATACTCTAA | Mutagenesis to generate *rad24‑650,652,654SSS→DDD* |
| O‑416 | rad24‑C‑Term‑A | GCGCCAGTTATCAGTGAGGCTCTTGCTGATGCTGATCTGGAAATACTCTAA | Mutagenesis to generate *rad24‑650,652,654SSS→AAA* |
| O‑417 | rad24‑S637D‑DpnI | GCGCCAGTTATCAGTGAGGATCTTGATGATGATGATCTGGAAATACTCTAA | Mutagenesis to generate *rad24‑S637D* |
| O‑418 | rad24‑S637A‑DdeI | GACGAGTCTCTATGTGAAATTCTGGCTCAGAGACAGCCGCGTAAAGCGCCA | Mutagenesis to generate *rad24‑S637A* |
| O‑467 | Y2H‑CAND‑rad24‑Delta(461‑527) | GACGAGTCTCTATGTGAAATTCTGGCTCAGAGACAGCCGCGTAAAGCGCCA | Mutagenesis to delete aa 461‑527 of Rad24 (*rad24‑ΔC1*) |
| O‑466 | rad24‑Delta(528‑594) | ACAAATTTAGTGATATAATGCTTGAAGACGAAGAAACTTC | Mutagenesis to delete aa 528‑594 of Rad24 (*rad24‑ΔC2*) |
| O‑464 | rad24‑Delta(595‑659) | TGATGATGGCTAACGACGATTAACTTTTTACTCTTTAAAT | Mutagenesis to delete aa 595‑659 of Rad24 (*rad24‑ΔC3*) |
| O‑506 | rad24‑D593A,D594A‑RC | ATTAAAAGAAGTTTCTTCGTCCTCGAGAGCGGCGTTAGCCATCATCACATTTCTTTC | Mutagenesis to generate *rad24‑593,594DD→AA* |
| O‑507 | rad24‑E598A,E599A‑RC | AATAGGGTCATCATTAAAAGAAGTTGCTGCGTCCTCGAGATCGTCGTTAGCCATCATCAC | Mutagenesis to generate *rad24‑598,599EE→AA* |
| O-609 | Rad24Delta(575‑601)Coil‑FOR | TTTAATGATGACCCTATTGTCGAT | Mutagenesis to delete aa 575‑601 of Rad24 (*rad24‑Δcoil*) |
| O-447 | rfa1-t11-REV | GGAAATCATGATCAAATTCTCTCTGTTGCTGTTGGCGCCATCAGATTTCTTGGTGTTATA | Mutagenesis to generate *rfa1-t11* |
