# Supplementary material for: Characterization of the Interaction between Rfa1 and Rad24 in Saccharomyces cerevisiae
Source: PLoS One. 2015 Feb 26;10(2):e0116512. doi: 10.1371/journal.pone.0116512 (PMC4342240; doi:10.1371/journal.pone.0116512)
Supplement: S4 Table — (DOCX) [file pone.0116512.s009.docx]

**Table S4. Other novel putative Rfa1-FLAB interactors**

| Rfa1 Interactor | Times Identified in Initial Screen | Times Identified in Damage Screen | Fusion Junction  (aa Residue #) | Description *^a^* |
| --- | --- | --- | --- | --- |
| Maf1 | 11 |  | 214 | Highly conserved negative regulator of RNA polymerase III |
| Axl2 | 5 | 1 | 522 | Integral plasma membrane protein |
| Ecm21 | 2 |  | 843 | Regulates endocytosis of plasma membrane proteins |
| Iml1 | 2 |  | 1151 | GTPase-activating protein required for non-nitrogen-starvation-induced autophagy |
| Mif2 | 2 |  | 462 | Orthologous to CENP-C and required for structural integrity of elongating spindles |
| Adh1 | 1 |  | 302 | Alcohol dehydrogenase required for the reduction of acetaldehyde to ethanol |
| Aim7 | 1 |  | 3 | Interacts with Arp2/3 complex to stimulate actin filament debranching and inhibit actin nucleation |
| Alr1 | 1 |  | 121 | Plasma membrane Mg^2+^ transporter |
| Apc4 | 1 |  | 27 | Subunit of the anaphase-promoting complex/cyclosome (APC/C) |
| Eht1 | 1 |  | 400 | Acyl-coenzymeA:ethanol O-acyltransferase |
| Fas1 | 1 |  | 621 | Beta subunit of fatty acid synthetase |
| Gap1 | 1 |  | 589 | General amino acid permease |
| Gpm3 | 1 |  | 189 | Homolog of Gpm1 phosphoglycerate mutase (function currently unknown) |
| Ldb19 | 1 |  | 376 | Alpha-arrestin involved in ubiquitin-dependent endocytosis |
| Mog1 | 1 |  | 123 | Conserved nuclear protein that interacts with GTP-Gsp1 to stimulate nucleotide release and is involved in nuclear protein import |
| Paa1 | 1 |  | 54 | Polyamine acetyltransferase that may be involved in transcription and/or DNA replication |
| Pkc1 | 1 |  | 236 | Protein serine/threonine kinase essential for cell wall remodeling during growth |
| Srp102 | 1 |  | 91 | Signal recognition particle receptor beta subunit |
| Ubp1 | 1 |  | 356 | Ubiquitin-specific protease capable of cleaving polyubiquitin chains |
| Vma21 | 1 |  | 45 | Integral membrane protein required for V-ATPase function |
| Vps29 | 1 |  | 163 | Subunit of the membrane-associated retromer complex essential for endosome-to-Golgi retrograde transport |
| Ydl180 | 1 |  | 478 | Unknown function; GFP-fusion localizes to vacuole |
| Ynr071c | 1 |  | 24 | Unknown function; putative aldose 1-epimerase |
| Med2 |  | 1 | 116 | Subunit of the RNA polymerase II mediator complex |
| Mhp1 |  | 1 | 235 | Microtubule-associated protein involved in microtubule organization |
| Opi1 |  | 1 | 90 | Transcriptional regulator of a variety of genes; involved in telomere maintenance |
| Tuf1 |  | 1 | 144 | Mitochondrial transcription elongation factor Tu |
| Yuh1 |  | 1 | 39 | Ubiquitin C-terminal hydrolase that cleaves ubiquitin-protein fusions to generate monomeric ubiquitin |

*^a^* All descriptions were obtained from the Locus Overview section in the *Saccharomyces* Genome Database ([www.yeastgenome.org](http://www.yeastgenome.org)) for each gene.
